# Supplementary material for: Evaluation of continuous quality improvement of tuberculosis and HIV diagnostic services in Amhara Public Health Institute, Ethiopia
Source: PLoS One. 2020 Mar 19;15(3):e0230532. doi: 10.1371/journal.pone.0230532 (PMC7081999; doi:10.1371/journal.pone.0230532)
Supplement: Attachment — Submitted filename: PONE-D-19-30524_R1 ebq.pdf [file pone.0230532.s002.pdf]

PLOS ONE

Evaluation of continuous quality improvement of tuberculosis and HIV diagnostic services in Amhara public health institute, Ethiopia

--Manuscript Draft--

**Manuscript Number:**	PONE-D-19-30524R1
**Article Type:**	Research Article
**Full Title:**	Evaluation of continuous quality improvement of tuberculosis and HIV diagnostic services in Amhara public health institute, Ethiopia
**Short Title:**	Quality improvement of tuberculosis and HIV diagnostic services in APHI
**Corresponding Author:**	Melashu Balew Shiferaw, MSc<br>Amhara Public Health Institute, Bahir Dar, Ethiopia<br>Bahir Dar, ETHIOPIA
**Keywords:**	Quality indicator, stock out, specimen rejection, TB, viral load, EID
**Abstract:**	Background: Unreliable laboratory results lead to unnecessary tests, procedures or treatments which may harm patient. Continuous quality improvement (CQI) is a useful objective tool to improve processes and services. The use of quality indicators that meet requirements for effectiveness is an important quality improvement tool. However, the quality of critical aspects of pre-examination, examination, and post-examination processes are not evaluated in Ethiopia including our setting. Hence, this study aimed to assess the performance of continuous quality improvement of TB and HIV laboratory tests in the Amhara Public Health Institute (APHI).<br>Methods: A cross-sectional study was conducted to evaluate the continuous quality improvement of advanced TB and HIV related laboratory tests in APHI from 01 January to 30 September 2019. HIV viral load, exposed infant diagnosis (EID), GeneXpert and TB culture quality indicators data were used as a quality improvement tool and evaluated in comparison to established targets. Data were extracted from excel database and record review of patient information, and entered and analyzed using SPSS V20 software.<br>Results: A total of 26,487 samples were received from 01 January to 30 September 2019. The overall specimen rejection rate was 0.43% (115/26,487). Specifically, viral load and TB culture had 0.43% and 1.14% rejection rates, respectively. The highest monthly rejection was documented for TB culture (5.3%) and viral load (2.4%) in September 2019. Centrifugation problems (46.1% [53/115]) and the use of wrong container (40.9% [47/115]) were the main reasons for the rejections. Moreover, EID test was interrupted for a total of 54 days and 22 days due to reagent stock out and equipment down time, respectively. Similarly, about 82% of viral load and 100% of the EID tests had long turnaround time (TAT) with an average of 24.1 and 29.3 days respectively in September 2019.<br>Conclusions: There were high rates of TB culture and viral load specimen rejection, and EID test interruptions. The trend of TAT of viral load and EID tests were longer than the targeted average TAT. Hence, training of sample collectors, functional equipment maintenance systems and supply chain management are recommended for continuous quality improvement.
**Order of Authors:**	Melashu Balew Shiferaw, MSc
	Abay Sisay
**Opposed Reviewers:**	
**Response to Reviewers:**	'Response to Reviewers'.<br>Dear editor, thank you so much for mailing the comments. We would like to thank the reviewer’s, really well appreciated critics and we learn a lot from the review, and also our work now has been improved much from their comments. The following is our point by point response:<br>Journal Requirements:<br>In the ethics statement in the manuscript and in the online submission form, please provide additional information about the patient records/samples used in your retrospective study. Specifically, please ensure that you have discussed whether all

Powered by Editorial Manager® and ProduXion Manager® from Aries Systems Corporation

data/samples were fully anonymized before you accessed them and/or whether the IRB or ethics committee waived the requirement for informed consent. If patients provided informed written consent to have data/samples from their medical records used in research, please include this information.
Response: thank you, now detailed ethics statement has been included in the manuscript and in the online submission form.

Reviewers' comments:

Reviewer #1:
Abstract:
Background:
• The focus of this study is continuous quality improvement (CQI) assessment using specific laboratory indicators but information on CQI not provided.
Response: Thank you, now it has been provided in the revised manuscript
• The established target was your laboratory specific and should be made clear as such in line 30 including specific target for each quality indicators.
Response: Thank you, it has been made clear now since include specific target for each quality indicators in table 1 of the new version manuscript. But, to include in the abstract part it became more detailed and out of word limit. So, table 1 and description in the method part is enough.
• Replace long waiting time with TAT and effect same correction throughout the text for uniformity and consistency of terminology.
Response: thank you, it has been replaced throughout the text
Methods:
• Line 26 through 30 should contain information of number of facilities and level of health care if such data is available. This would help for another level of analysis to improve the work further. How was the data collected, reviewed cleaned and analyzed was not stated.
Response: thank you, now we have stated how the data was collected, reviewed cleaned and analyzed. However, information of number of facilities and level of health care data was not available to include as suggested.
• Line 38 stated that ''EID had a long waiting time in September 2019''. What specific time are you referring to?. Include the exact data for the TAT.
Response: thank you, now it has been described. The specific time was 29.3 days as described in the new version manuscript.

Introduction:
• The authors may wish to consider this statement Continuous quality improvement (CQI) is a useful objective tool to improve processes and services and linking it with line 45 through 48 and ensuring effective flow and synchronization. Pre-analytical error and Post-analytical error data are abundant and well documented and would be important to include them in the introduction.
Response: : thank you, pre-analytical error and Post-analytical error data has been included them in the introduction

• Line 54 replace ''equipment down'' with equipment down time and do same throughout text and include internal quality control (IQC) after proficiency testing in line 55.
Response: thanks, now it has been corrected as the reviewer's advice.
• Replace "has been magnified more'' with would become magnified in line 66 and expunge such and of in line 74 and 76 respectively.
Response: thanks, now it has been corrected as the reviewer's advice.
Materials and Methods:
Authors may wish to consider subheading using the following guide for easy readability:
1. Study Design and ensure the actual study design was well stated since data was retrospective
Response: thanks, now it has been corrected
2. Setting should be well describe including longitude and latitude of the study facility
Response: Now it has been well described as shown in the setting part of the method section
3. Data Collection
Response: thanks, now it has been revised

	4. Quality indicators and objective of each<br>Response: thanks, now it has been revised and we include it together with data collection part<br>5. Data review, cleaning and data Analysis<br>Response: thanks, now it has been revised<br>6. Ethical Approval or Ethical Consideration<br>Response: thanks, now it has been revised<br>7. Table for quality indicator and threshold or target for the laboratory<br>Response: Thank you, now the table has been included as the reviewer's advice.<br>Line 91 replace maintained with repair or fix and do same for gexpert with GeneXpert in line 93.<br>Response: thanks, now it has been corrected<br>Please define all abbreviations such as RR, RRF, RRF and DBS in text before they can be use.<br>Response: thanks, now it has been revised<br>What of Internal quality control monitoring threshold or target for qualitative data such as viral load testing?<br>Response: Thanks, it is 100%. It is described in table 1 of the new version manuscript.<br>Results Section:<br>• You may wish to consider including characteristics of facilities and various level of health cares including ownership (private and public) in the result section. Include percentage in parenthesis for each absolute number from lie 132 through 137.<br>Response: Thanks, now we have included percentage in parenthesis for each absolute number from line 132 through 137. However, we couldn't get data for characteristics of health facilities to include as the reviewers recommendation.<br>• Ensure you do not repeat results in tables for example use term like about half or close to half or one-third etc.<br>Response: Thank you for the comment; it has been revised according to the reviewers advice.<br>• Between line 160 and 167 ensure that text are presented as they are arrange in heading and remove was in line 164 after rate. Replace March (2.8%) to June (5.0%) in 2019 with what you have in line 164.<br>Response: Thank you, now corrections have been made based on the reviewer comments.<br>• Please replaced waiting time with TAT with waiting time throughout text see Line 175 through 190.<br>Response: Thank you, now waiting time has been replaced with TAT in the revised version manuscript.<br>• What of GeneXpert Data?<br>Response: Now, we specified in the result section under TAT in addition to table 5<br>• In line 179 replace interestingly with However and expunge was in 181 after TAT, include 2019 after May in line 181 and same in line 189 after February.<br>Response: Thank you, now it has been corrected<br><br>Discussion:<br>• Authors may wish to consider major findings summary as the first paragraph and discussion of results chronologically as presented in result section in next paragraphs thereafter<br>Response: Thanks, now we add summary of results in the first paragraph of the discussion section as the reviewer's advice.<br>• Include period of months in line 193 and replace This finding and is in line 195 with Those findings and was.<br>Response: Now, it has been corrected<br>• Where was 0.26% reported by Cao et al., in line 197 and modify sample recollection with repeat specimen collection in line 197 and any other place recollection of sample appear in text<br>Response: Thanks, it was in Huston, United States. And recollection has been modified based on the reviewer's advice.<br>• In line 199, include compromise before patient safety and modify resources wastes to waste resources.<br>Response: Thank you very much; it really helps us to improve our paper. And it has been modified accordingly.<br>• In line 203 replace sample collectors with Phlebotomy and do so through text and replace sole in line 205 with avoid and include nearly half in line 2016 before 46.1%

	and include specimen before containers in line 207<br>Response: Thank you; all recommendations have been corrected in the new version manuscript except Phlebotomy that it better to use as it's, because the term phlebotomy more related to blood collections, but in our study, we have more than blood sample like sputum.<br>• In line 209 include for a period of after interrupted and remove in the study after month.<br>Response: Thank you; now it has been modified<br><br>• The sentence in line 212 to 213 require modification and done after studies should be expunge<br>Response: Thank you; now it has been modified<br>• In line 222 replace is with was and in line 223 remove and replace observed or recorded with seen and line 224 that the should be replace with where and include period after months in 227.<br>Response: Now, it has been corrected<br><br>Conclusion:<br><br>Effect on necessary correction pointed out in discussion in conclusion include specimen in line 231 before container and time after down and phlebotomy in line 233 to replace samples collectors and include improved in line 234 after and before supply.<br>Response: Now, it has been corrected<br>Tables and Figures<br>• Table 1 Epert TB should read GeneXpert TB and include Abbreviations Definition under table<br>Response: Now, it has been corrected<br>• Table 2 inlcude n(%) for each test and include Abbreviations and Definition<br>Response: Response: Now, it has been corrected<br>• Include figure title under each Chart for Line Graph for Fig 1 and Fig 2 respectively<br>Response: Thank you for the comment. The journal requirement describes to provide figures in separate and title to include in the main manuscript. That is why we didn't put under each figure. The editor may put it under the graph during publication.<br><br><br>Reviewer #2:<br>Introduction<br>1. Lines 54-58: You mention that "recommended quality indicators for the post-analytical phase are turnaround time (TAT), the percentage of incorrect laboratory test reports, and notification of critical results," yet you do not measure or report on incorrect laboratory results or notification of critical results. Do you have these data? If not, consider adding to the limitations.<br>Response: Thank you, comment accepted and it has been described as limitation at the end of the discussion part<br>2. Lines 59-62: Confusing sentence, consider revising<br>Response: Thank you, now it has been revised to avoid the confusion.<br><br>3. Lines 68-71: The statistics for MRD/RR-TB and ART use seem out of place here and hard to contextualize in terms of laboratory testing requirements. Would be more useful to know – at a national level – how many patients require each of these tests annually.<br>Response: Thank you. The statistics described was to give background information how much of the patients suffer from HIV and TB related health problems at national level including our setting. All of the listed statistics require quality assured laboratory results. So, we believe this information is important and has not been deleted.<br><br>Methods<br>4. Line 111: Should be "Oneworld Accuracy", please ensure correct spelling and capitalization. Also suggest citing<br>Response: Thank you, now it has been corrected and cited.<br><br>5. Line 93: GeneXpert spelled incorrectly.<br>Response: Thank you, now it has been corrected<br><br>6. Lines 89: How does the indicator "test interruption" differ from equipment downtime

	and stock out?<br>Response: Thank you, it differs because the lab uses back up referring laboratories to avoid test interruption when there is either equipment downtime or stock out.<br>7. Line 95: Unclear to reader what “Besides, reagent stock out status was set to be zero” means.<br>Response: now it has been modified. It means no reagent stock out was set as a quality indicator by APHI.<br><br>8. Line 102: Suggest rephrasing to say, “sample received 5+ days after collection…”<br>Response: Thank you, now it has been rephrased as the reviewer’s advice<br><br>9. Line 104, “old” plasma samples – please quantify what how old is “old”<br>Response: Thanks, it was said old plasma samples if it was delayed >5 days after collection when transported to APHI at 2-8oC. Now it has been included in the new manuscript.<br><br>10. Lines 109-114, please describe in more detail what proficiency testing entails.<br>Response: Now it has been more entailed and included in the new version manuscript.<br>11. Lines 112-118: How were these targets (PT 80%, contamination <5%, error <5%, TAT for each test) established?<br>Response: Thanks, now it has been included in table 1 of the new version manuscript<br><br>Results<br>1. The presentation of results is difficult to follow. I would suggest presenting the all of results for each test separately and then moving on to the next test, keeping the same format and organization for the presentation of results of each test. For example, discuss TB culture first and talk about:<br>a. Pre-examination indicators:<br>i. total number of samples for culture TB<br>ii. rejection rate for culture TB and reasons for rejection<br>b. Examination phase indicators:<br>i. Test interruption for culture TB & reasons<br>ii. Error/contamination rates and reasons for culture TB<br>c. Post examination phase indicators:<br>i. TAT for culture TB<br>Then repeat this sequence of results presentation for GeneXpert, EID, and VL.<br>Response: Thank you for the comments. But when we tried to rearrange based on the suggested sequence of results, it became more complicated and difficult to describe repeated quality indicators. So, we prefer the current sequence of results presentation.<br>2. The inclusion of data per month complicates the results, without adding much benefit, since there does not seem to be a temporal trend in indicators. I would suggest removing references to months when specific rates were achieved. Instead, the results could be simplified by first giving the average across the entire study period and then giving the low to high range, rather than specify the value per month.<br>a. For example, Lines 165-167 could be simplified to “Regarding TB GeneXpert, the average error rate over the study period was XX%, with monthly error rates ranging from 0%-4%.”<br>Response: now it has been simplified based on the reviewer’s advice.<br><br>3. No examination phase measures for HIV viral load testing are discussed.<br>Response: Thank you, it has been already discussed in the result section under proficienc testing performance stating “All of the tests participated in the PT program qualified the minimum requirement that the passing mark was 80%. Specifically, viral load, EID and GeneXpert had 100% performance as evaluated in April 2019”. But IQC was not discussed since we didn’t evaluate it and we put as limitation in the new version manuscript.<br><br>4. average TAT across the study period was not specified for each test, rather, monthly rates were given, which unnecessarily complicates the results.<br>Response: Thank you, now it has been included in the result section and in table 5.<br><br>5. TAT for GeneXpert RR tests are not specified, other than “good”.<br>Response: Now, it has been specified in the new version manuscript

Powered by Editorial Manager® and ProduXion Manager® from Aries Systems Corporation

	6. Tables 4 and 5. What is the difference between turnaround time and waiting time?<br>Response: Thank you so much, comments help us to improve the paper. Table 4 describes the proportion of out of TAT patient results in APHI, and Table 5 describes the average TAT of tests. In the new version manuscript, the title of table 4 has been modified as “Proportion of out of turnaround time of patient results in APHI reference laboratories, January to September 2019”<br><br>7. Table 4: I’d suggest a title that makes it clearer that the numbers presented are rates of samples that achieved TAT targets. As is, that is not clear.<br>Response: Thank you the title now has been cleared. Yes, rates of samples that achieved TAT targets<br><br>Discussion<br>• The authors miss an opportunity to discuss recommendations to address the gaps that their data highlights. The only recommendation offered discusses the need for training to address sample rejection issues (lines 202-206). While important, this will not help with machine down, stockouts and long turnaround times – what can be done to improve indicators for these?<br>Response: Thank you so much, very important view and help to improve our work. Now, recommendations have been included to address the gaps highlighted.<br><br>• Please discuss the 2 months where no EID tests were conducted – what happened to the EID samples during the period: were they sent to other labs? Were they held on to and processed once the machine was repaired and reagents replenished – if so, this could explain the increased sample volume and TAT observed in May?<br>Response: Thank you, the 2 months EID interruption was due to the national stock out occurred in Ethiopia that all of the labs in Ethiopia were not able to test EID. It was impossible to send samples to other laboratories. In addition, equipment was failed in April for seven days as explained in the result section. Maintenance was done after that. No, these issues have been explained in the discussion section.<br><br>• Line 221-222: “About 82% of viral load tests and 100% of the EID tests had an average TAT of 45.6 days in September 2019.”<br>Response: Thank you so much, now it has been corrected as “About 82% of viral load tests and 100% of the EID tests had out of targeted TAT in September 2019”.<br><br>• Suggest adding a limitations section.<br>Response: thanks, now limitation section has been included in the new version manuscript.<br><br>General comments<br>1. Please define all acronyms at first use. Some that were not accurately defined include: MDR/RR-TB (line 68); IFRR/RRF (line 96); DRTB (line 98), MGIT (line 152), LJ culture (line 154)<br>Response: thanks, now it has been revised<br>2. Please be sure to write GeneXpert with correct capitalization pattern<br>Response: thanks, now it has been revised
**Additional Information:**	
**Question**	**Response**
**Financial Disclosure**<br><br>Enter a financial disclosure statement that describes the sources of funding for the work included in this submission. Review the submission guidelines for detailed requirements. View published research articles from *PLOS ONE* for specific examples.<br><br>This statement is required for submission	The author(s) received no specific funding for this work.

and **will appear in the published article** if the submission is accepted. Please make sure it is accurate.<br><br>**Unfunded studies**<br>Enter: *The author(s) received no specific funding for this work.*<br><br>**Funded studies**<br>Enter a statement with the following details:<br>• Initials of the authors who received each award<br>• Grant numbers awarded to each author<br>• The full name of each funder<br>• URL of each funder website<br>• Did the sponsors or funders play any role in the study design, data collection and analysis, decision to publish, or preparation of the manuscript?<br>• **NO** - Include this sentence at the end of your statement: *The funders had no role in study design, data collection and analysis, decision to publish, or preparation of the manuscript.*<br>• **YES** - Specify the role(s) played.<br><br>* typeset	
**Competing Interests**<br><br>Use the instructions below to enter a competing interest statement for this submission. On behalf of all authors, disclose any competing interests that could be perceived to bias this work—acknowledging all financial support and any other relevant financial or non-financial competing interests.<br><br>This statement **will appear in the published article** if the submission is accepted. Please make sure it is accurate. View published research articles from *PLOS ONE* for specific examples.	The authors have declared that no competing interests exist.

**NO authors have competing interests**<br><br>Enter: *The authors have declared that no competing interests exist.*<br><br>**Authors with competing interests**<br><br>Enter competing interest details beginning with this statement:<br><br>*I have read the journal's policy and the authors of this manuscript have the following competing interests: [insert competing interests here]*<br><br>* typeset	
**Ethics Statement**<br><br>Enter an ethics statement for this submission. This statement is required if the study involved:<br><br>• Human participants<br>• Human specimens or tissue<br>• Vertebrate animals or cephalopods<br>• Vertebrate embryos or tissues<br>• Field research<br><br>Write "N/A" if the submission does not require an ethics statement.<br><br>General guidance is provided below. Consult the submission guidelines for detailed instructions. **Make sure that all information entered here is included in the Methods section of the manuscript.**	Regarding ethical issue, the data were not collected from patients directly since the specimens were collected from the peripheral health facilities and sent to the testing center through a referral network. Before data collection, ethics approval and official permission letter (Reference number: HRTT/03/139/2018) was obtained from the APHI research and technology transfer directorate to use patient records from the APHI excel database, quality indicator reports and record review of different quality related records in the laboratory. And the Research and Technology Transfer Directorate waived the requirement for informed consent.

Powered by Editorial Manager® and ProduXion Manager® from Aries Systems Corporation

**Format for specific study types**<br><br>**Human Subject Research (involving human participants and/or tissue)**<br>• Give the name of the institutional review board or ethics committee that approved the study<br>• Include the approval number and/or a statement indicating approval of this research<br>• Indicate the form of consent obtained (written/oral) or the reason that consent was not obtained (e.g. the data were analyzed anonymously)<br><br>**Animal Research (involving vertebrate animals, embryos or tissues)**<br>• Provide the name of the Institutional Animal Care and Use Committee (IACUC) or other relevant ethics board that reviewed the study protocol, and indicate whether they approved this research or granted a formal waiver of ethical approval<br>• Include an approval number if one was obtained<br>• If the study involved *non-human primates*, add *additional details* about animal welfare and steps taken to ameliorate suffering<br>• If anesthesia, euthanasia, or any kind of animal sacrifice is part of the study, include briefly which substances and/or methods were applied<br><br>**Field Research**<br><br>Include the following details if this study involves the collection of plant, animal, or other materials from a natural setting:<br>• Field permit number<br>• Name of the institution or relevant body that granted permission	
**Data Availability**<br><br>Authors are required to make all data underlying the findings described fully available, without restriction, and from the time of publication. PLOS allows rare exceptions to address legal and ethical concerns. See the PLOS Data Policy and FAQ for detailed information.	Yes - all data are fully available without restriction

A Data Availability Statement describing where the data can be found is required at submission. Your answers to this question constitute the Data Availability Statement and **will be published in the article**, if accepted.<br><br>**Important:** Stating 'data available on request from the author' is not sufficient. If your data are only available upon request, select 'No' for the first question and explain your exceptional situation in the text box.<br><br>Do the authors confirm that all data underlying the findings described in their manuscript are fully available without restriction?	
**Describe where the data may be found in full sentences. If you are copying our sample text, replace any instances of XXX with the appropriate details.**<br><br>• If the data are **held or will be held in a public repository**, include URLs, accession numbers or DOIs. If this information will only be available after acceptance, indicate this by ticking the box below. For example: *All XXX files are available from the XXX database (accession number(s) XXX, XXX.*).<br>• If the data are all contained **within the manuscript and/or Supporting Information files**, enter the following: *All relevant data are within the manuscript and its Supporting Information files.*<br>• If neither of these applies but you are able to provide **details of access elsewhere**, with or without limitations, please do so. For example:<br><br>*Data cannot be shared publicly because of [XXX]. Data are available from the XXX Institutional Data Access / Ethics Committee (contact via XXX) for researchers who meet the criteria for access to confidential data.*<br><br>*The data underlying the results presented in the study are available from (include the name of the third party*	All relevant data are within the manuscript.

and contact information or URL).<br>• This text is appropriate if the data are owned by a third party and authors do not have permission to share the data.<br><br>* typeset	
Additional data availability information:	

Powered by Editorial Manager® and ProduXion Manager® from Aries Systems Corporation

Dear Sir/Madam,

I am writing to submit our manuscript entitled "**Evaluation of continuous quality improvement of tuberculosis and HIV diagnostic services in Amhara public health institute, Ethiopia**" for consideration of publication in *PLOS ONE* journal. This study investigated laboratory quality improvement practice in APHI. Our findings could be applied in the health service delivery; they are likely to be of great interest to researchers, health practitioners, planner, and decision maker who read your journal. *PLOS ONE* journal is an open access journal; it allows us to share our finding to the wide global community. All the authors approved the manuscript and this submission.

Kind regards,

Melashu Balew Shiferaw

Corresponding author

Manuscript Click here to access/download;Manuscript;Manuscript R1.docx

Evaluation of continuous quality improvement of tuberculosis and HIV diagnostic services in Amhara public health institute, Ethiopia

Melashu Balew Shiferaw[1]*, Abay Sisay Misganaw[2]

[1]Research and Technology Transfer Directorate, Amhara Public Health Institute, P.O.Box 447, Bahir Dar, Amhara, Ethiopia

[2]Department of Medical Laboratory Sciences, College of Health Sciences, Addis Ababa University, Addis Ababa, Ethiopia

*Corresponding Author

E-mail: bmelashu@gmail.com (MBS)

Abstract

Background: Unreliable laboratory results lead to unnecessary tests, procedures or treatments which may harm patient. Continuous quality improvement (CQI) is a useful objective tool to improve processes and services. The use of quality indicators that meet requirements for effectiveness is an important quality improvement tool. However, the quality of critical aspects of pre-examination, examination, and post-examination processes are not evaluated in Ethiopia including our setting. Hence, this study aimed to assess the performance of continuous quality improvement of TB and HIV laboratory tests in the Amhara Public Health Institute (APHI).

Methods: A cross-sectional study was conducted to evaluate the continuous quality improvement of advanced TB and HIV related laboratory tests in APHI from 01 January to 30 September 2019. HIV viral load, exposed infant diagnosis (EID), GeneXpert and TB culture quality indicators data were used as a quality improvement tool and evaluated in comparison to established targets. Data were extracted from excel database and record review of patient information, and entered and analyzed using SPSS V20 software.

Results: A total of 26,487 samples were received from 01 January to 30 September 2019. The overall specimen rejection rate was 0.43% (115/26,487). Specifically, viral load and TB culture had 0.43% and 1.14% rejection rates, respectively. The highest monthly rejection was documented for TB culture (5.3%) and viral load (2.4%) in September 2019. Centrifugation problems (46.1% [53/115]) and the use of wrong container (40.9% [47/115]) were the main reasons for the rejections. Moreover, EID test was interrupted for a total of 54 days and 22 days due to reagent stock out and equipment down time, respectively. Similarly, about 82% of viral load and 100% of the EID tests had long turnaround time (TAT) with an average of 24.1 and

29.3 days respectively in September 2019.

Conclusions: There were high rates of TB culture and viral load specimen rejection, and EID test interruptions. The ~~trend of~~ TAT of viral load and EID tests were longer than the targeted average TAT. Hence, training of sample collectors, functional equipment maintenance systems and supply chain management are recommended for continuous quality improvement.

Keywords: Quality indicator, stock out, specimen rejection, TB, viral load, EID

Introduction

Continuous quality improvement (CQI) is a useful objective tool to improve processes and services. In modern clinical medicine, laboratory tests play an important role in diagnosing, monitoring, and evaluating patient outcomes. Hence, the implementation of performance measurements to evaluate the pre-analytical, analytical and post-analytical stages of the total testing process is therefore needed to maximize the overall testing cycle and the quality of patient care [1].

Pre-analytical error and Post-analytical error data are well documented. Occurrence of the errors at the pre- and post-analytical phases currently appears to be more vulnerable than the analytical phase [2]. Studies revealed that 46%–68.2% of laboratory errors predominated in the pre- analytical phase, and 18.5%–47% errors recorded in the post-analytical phase of the laboratory testing process [3-6].

The use of quality indicators that meet requirements for effectiveness and harmonization is an important quality improvement tool [7]. It can measure how well an organization meets the needs and requirements of users and the quality of all operational processes. Monitoring quality

indicators in daily work can reduce laboratory errors and risk to patient safety by identifying problems in all phases of the laboratory process, allowing their correction [8].

Stock out of reagents, equipment down time, and sample rejection rates are common pre-analytical phase quality indicators, whereas the performance of proficiency testing (PT) in external quality assessment (EQA) program, internal quality control (IQC) and contamination or error rates are analytical phase indicators. ~~Besides,~~ minimum recommended quality indicators for the post-analytical phase are turnaround time (TAT), the percentage of incorrect laboratory test reports, and notification of critical results [8, 9].

Laboratories should deliver accurate, reliable and timely results to customers. When the results are compromised in quality and/ or delayed, it could have an impact on patient management. As a result, the clinician may interpret the results as actionable which, in turn, can lead to unnecessary tests, procedures or treatments which may result in patient harm [10].

According to the World Health Organization (WHO), drug resistance TB and HIV are global challenges that need quality-assured laboratory tests for better treatment options and control of the burden [11]. In addition to the drug resistance burden, if there is poor quality laboratory service, the problem ~~would be~~ magnified.

In Ethiopia, where the estimated proportion of TB cases with Multidrug resistant/ rifampicin-resistant tuberculosis (MDR/RR-TB) was16% among previously treated cases and reported a total of 428,472 HIV patients receiving antiretroviral therapy in 2018 [12, 13], laboratory tests are performed to control drug resistance and monitor disease progression. TB culture is performed to identify treatment failures, exposed infant diagnosis (EID) to identify HIV status in exposed infants, and viral load to monitor confirmed viral failure and estimate disease

progression. However, the quality of critical aspects of pre-examination, examination and post-examination processes of TB and HIV related laboratory services are not evaluated including ~~our setting~~. Hence, this study aimed to ~~assess the performance of continuous quality improvement~~ using nine months quality indicators data in the Amhara Public Health Institute (APHI).

Materials and methods

Study design

A cross-sectional study was conducted to evaluate the continuous quality improvement of advanced TB and HIV related laboratory tests in APHI from 01 January to 30 September 2019.

Study setting

APHI is a government public health institute located in Bahir Dar town, Ethiopia at 11°60′N latitude and 37°37′E longitude. The institute has three main directorates (Laboratory, Public Health Emergence management, and research and technology transfer). It provides laboratory diagnostic services including TB (culture for treatment failure, and GeneXpert rifampicin resistance), EID and viral load diagnosis to identify ~~drug resistance for TB,~~ HIV status of infants and confirm~~ed~~ antiretroviral failure, respectively. These TB and HIV related tests are requested from referring peripheral health facilities in Amhara region ~~that have been networked~~ through a sample referral network. APHI is used as a reference testing center for more than 150 health facilities. All the laboratory tests investigated in APHI including HIV viral load, EID, TB culture and GeneXpert have been accredited by the Ethiopian National Accreditation Office (ENAO) in complying with the ISO 15189:2012 standard since October 2018 [14].

Data collection, and quality indicators and objective of each

In APHI, quality indicators were established to monitor the laboratory performance. EQA PT, contamination rate, error rate specimen rejection rate, TAT, IQC performance, stock out, service interruption and equipment down time were the APHI quality indicators (Table 1). Each target was established by reviewing a minimum of 3 months of data at APHI and also reviewing international literatures.

All of the requested MDR TB, EID and viral load requested tests were included consecutively in the study. Data were collected using a record review of monthly quality indicator reports from January to September 2019. The established quality indicators at the pre-examination phase were equipment down time, stock out, test interruption, and specimen rejection rate.

Equipment down time was set as a quality indicator of APHI virology and bacteriology reference laboratories. All of the equipment should be repaired within 5days if there is a failure. Abbott 2000SP and Abbott 2000RT were used for extraction and detection of ribonucleic acid (RNA) of EID and HIV viral load tests. Furthermore, GeneXpert machine for TB RR testing. TB culture needs 35-37°C incubators. So, we reviewed maintenance records and books of life for each of the equipment in the study period. Besides, no reagent stock out was set as a quality indicator by APHI. Reagent inventory records such as bin card, internal facility request and resupply form (IFRR), and report and requisition form (RRF) were reviewed to confirm the occurrence of stock out.

Excel database maintained in the central reception was used to review specimen rejections. Sputum for drug resistant tuberculosis (DRTB) /MDRTB, dried blood spot for EID and blood plasma for viral load samples were submitted at APHI central reception. The entire submitted patient samples were evaluated for sample quality based on pre-set sample rejection criteria.

Sputum samples were rejected when there was labeling error, leaking specimen container, sample volume less than 2ml, sample received 5+ days after collection, use of the wrong container, specimen containing blood and food ruminants, and temperature exceeding 8°C. The plasma sample was rejected if samples were collected using the wrong container, not-centrifuged, hemolyzed, old (if it was delayed >5 days after collection when transported to APHI at 2-8°C), insufficient volume, labeling error and if not transported below 8°C temperature. ~~Besides,~~ dried blood spot (DBS) was rejected when there was hemolysis, insufficient volume of spots, clotting and labeling error. Based on the APHI policy, rejected samples should be communicated to customers and another sample should be recollected for the requested test. The targeted specimen rejection rate was less than 2%.

Proficiency testing performance, contamination rate, error rates, and internal quality control were used for examination phase quality indicators. All of the evaluated tests (TB culture, GeneXpert, EID, and viral load) participated in proficiency testing from Oneworld Accuracy, which is an approved EQA PT provider. Panels of samples prepared from the PT provider and sent to APHI. In APHI, the PTs were diagnosed as patient samples and results were submitted online on the Oneworld Accuracy website. Proficiency testing performance of 80% and above was the pass mark acceptable results [15]. ~~Regarding contamination rate and error rates,~~ less than 5% target was set as quality indicators by APHI. Applicable test records were reviewed to evaluate examination phase performances.

Moreover, turnaround time was a post-examination phase quality indicator that 90% of tests should be released within defined turnaround ~~time. TAT of 10 days was targeted for viral load and EID tests. TB culture test TAT was set to be 48 days for each negative result and 64 days for each positive result. Besides, GeneXpert test targeted TAT was 2 days.~~ The TAT was calculated

from login and log out of records of each test achieved at the excel database of the central reception. ~~Hence,~~ the TAT of each test was evaluated based on established targets of the institute (Table 1).

Data review, cleaning and data Analysis

The data were entered and analyzed using SPSS V20 software. Selected quality indicators were compared with the defined target and rated as good if complied or not good if not complied with the established target.

Ethical Consideration

~~Regarding ethical issue,~~ the data were not collected from patients directly since the specimens were collected from the peripheral health facilities and sent to the testing center through a referral network. Before data collection, ethics approval and official permission l~~etter~~ (Reference number: HRTT/03/139/2018) was obtained from the APHI research and technology transfer directorate to use patient records from the APHI excel database, quality indicator reports and record review of different quality related records in the laboratory. ~~And the~~ Research and Technology Transfer Directorate waived the requirement for informed consent.

Results

Test statistics and specimen rejection

In Amhara Public Health Institute, a total of 26,487 HIV and TB related test requests with samples were received from 01 January to 30 September 2019. Of which, 26372 (99.6%) had acceptable quality. Among the acceptable samples, 24517 (93.0%) were blood plasma samples for viral load, 862 (3.3%) were dried blood spot samples for EID, 694 (2.6%) were sputum

samples for TB culture and 299 (1.1%) were sputum samples for TB GeneXpert tests (Table 2).

A total of 115 were rejected from January to September 2019. The overall specimen rejection rate was 0.43% (115/26,487). Specifically, viral load, TB culture and TB GeneXpert specimens had 0.43% (106/24623), 1.14% (8/702) and 0.33% (1/300) rejection rates, respectively. High monthly sample rejection was documented for TB culture and viral load in September 2019 with 5.3% (5/95) and 2.4% (61/2557) rejection rates, respectively (Table 3).

Centrifugation problems (46.1% [53/115]) and the use of wrong container (40.9% [47/115]) were the main reasons for specimen rejection. Viral load specimens were due to centrifugation problem (53/106), use of wrong container (47/106), insufficient volume (5/106) and hemolysis (1/106). Moreover, the old samples (5/8), use of a wrong container (1/8), insufficient volume (1/8) and uncontrolled temperature (1/8) were causes of rejection for TB culture samples. GeneXpert sample was rejected because of insufficient volume. During specimen rejection, there was proof of communication records about rejected samples. However, there was no evidence of receipt of another sample for the requested tests.

Test interruption

In this study, test interruption was used as a quality indicator. Mycobacteria growth indicator tube (MGIT) TB tests were stocked out. Even if the backup laboratory was assigned, it was impossible to manage due to the issue is national stock out problem and only the Lowenstein-Jensen (LJ) culture method was used. EID testing service was continuously interrupted. Reagent stock out (HIV1 qualitative control stocked out for two months: 05 March to 28 April 2019) and equipment down time (Abbott 2000rt was failed for seven days in March 2019 and Abbott 2000sp was failed for 15 days since 27 September 2019) were the main causes for the EID test

interruption (Table 4).

Genexpert error rates and TB culture contamination rates

In the Amhara Public Health Institute, the error rate was used as a quality indicator to follow TB GeneXpert tests and contamination rate as used to follow TB culture. Both of the indicators were targeted to be less than 5%. Interestingly, all of the monthly error rates and contamination rates reported were within the targeted limit that had been established by the institute. TB culture contamination rate increased from March (2.8%) to June (5.0%) in 2019. Then, it was continuously decreased up to the end of August 2019 (1.0%). Regarding TB GeneXpert, the average error rate over the study period was 1.4%, with monthly error rates ranging 0% - 4.0% (Fig. 1 and 2).

Performance of proficiency testing

The laboratories participated in April 2019 in the international PT from Oneworld Accuracy, which is an approved PT provider. All of the tests participated in the PT program qualified the minimum requirement that the passing mark was 80%. Specifically, viral load, EID and GeneXpert had 100% performance as evaluated in April 2019. Besides, TB culture PT performance was 80%, which fulfilled the minimum passing mark.

Turnaround times

The majority of the tests conducted for TB related laboratory diagnosis had good TAT. TB culture tests were released within the targeted TAT except in September 2019. In September, about 11% of the TB culture tests were not released with the targeted TAT. Minimum and maximum average TAT was 43.3 and 65.7 days documented in February and August 2019,

respectively. TB GeneXpert tests ~~were relatively had good~~ TAT in March, June and July 2019. However, the average TAT across the study period was 2.3 days with a range of 1.0 to 4.0 days.

Regarding the viral load tests, the average TAT over the study period was 16.5 days, ranging 7.6 to 24.1 days. The TAT improved from January to July 2019 except May 2019 with 18.6% of requested viral load tests out of the defined TAT. Furthermore, the TAT was longer in August and in September 2019 ~~that~~ 12.6%, and 81.5% of viral load tests were delayed, respectively.

EID tests were not done in January, March, and April. ~~However, the TAT for EID was affected more from time to time. For instance, about 35% of the EID tests were delayed in May 2019. Then, 50.6%, 67.4%, 100.0% and 100% of the tests were out of targeted TAT in June, July, August and September 2019, respectively. Over the nine months that the EID test functional,~~ the minimum TAT was 23.4 days occurred in February 2019 and the maximum TAT was 45.6 days documented in May 2019 (Table 5 and6).

Discussion

~~In this study, 0.45% of the specimens were rejected. EID and liquid TB culture (MGIT method) tests were interrupted over two months in the study period. Similarly, reagent stock out and equipment down time were occurred this period. About 82% of viral load tests and 100% of the EID tests had an average TAT of 45.6 days in September 2019.~~

In this study, the overall specimen rejection rate was 0.43%. Specifically, it was 1.14% for TB culture and 0.43% for viral load tests from January to September 2019. Surprisingly, high monthly sample rejection was documented for TB culture and viral load in September 2019 with 5.3% and 2.4% rejection rates respectively. Those findings were higher compared to the 2% monthly quality indicator target established by the institute. Also, the finding is higher than the

specimen rejection of 0.26% reported by Cao et al in Huston, United States [16]. This leads to a high rate of repeat specimen collection, delay in result availability and high rate of specimen/ test abandonment [17, 18]. Besides, it will have a delay of care, compromise patient safety, waste resources, patient discomfort, and potential patient complications especially if there are critical values [19-21]. In the setting, there was no evidence of repeat collection of another sample for the requested tests; rather there was proof of communication records about rejected samples. Hence, the laboratory should proactively take actions such as training of sample collectors on how to collect sufficient samples and process centrifugation of collected samples, and use appropriate sample collection containers in order to avoid rejection of samples referred to the institute from peripheral facilities in Amhara region since our data support that nearly half (46.1%) of the specimens were rejected due to centrifugation problem and 40.9% of the samples were rejected because of use of wrong specimen containers.

Without reliable, affordable, and portable devices, regular testing remains a challenge [22]. In this study, the EID testing service was continuously interrupted for a period of over two months in addition to the interruption of liquid TB culture (MGIT method) testing. In this period, it was impossible to send samples to other back up laboratories because there was reagent stock out at the national level that all of the laboratories in Ethiopia couldn't do the testing. Likewise, about 93% of the testing centers in Ghana had shortage of EID reagents [23]. Similarly, equipment down time was the main cause of test interruptions. Studies in Addis Ababa Ethiopia and in Malawi also revealed that the major reported factors affecting the provision of quality services were a shortage of resources and equipment failure [24, 25]. As a quality improvement plan, the laboratory could reduce interruption of tests through rapid implementation of equipment maintenance service agreement, making back up referring laboratory functional, and building

maintenance workshop when there is equipment failure. In addition, the institute should establish a system for international bid for advanced laboratory test reagents and supplies when these items stocked out at national level.

An efficient laboratory workflow ensures that specimens received in the laboratory are tested within the established laboratory turnaround time and results are returned to health care providers and their patients on time. However, specimen backlogs can occur when there is suboptimal workflow in the laboratory network as the result of poor sample quality, reagent or consumable stock out, and equipment breakdown [26]. In this study, the TAT of viral load and EID tests were longer. About 82% of viral load tests and 100% of the EID tests had out of targeted TAT in September 2019. The finding was higher than a study done in Myanmar where long TAT was observed in 69% of the participants [27], and in Kenya and Malawi where EID and viral load tests lasted average TAT of 24.7 and 8 days, respectively [28, 29]. This could be due to stock-outs of reagents and maintenance issues with the automated PCR testing equipment since our data showed reagent stock out of EID in about 54 days and equipment down time for a total of 22 days in the nine months from January to September 2019. In general, the ~~trend of~~ TAT for viral load and EID showed ~~more delay up to~~ the end of September 2019. ~~Hence, APHI laboratory should work on equipment management and stocking issues to provide timely laboratory result to customers.~~

Limitations

~~Reader should be into consideration the following limitation while inferring our result. We only measure TAT to evaluate for the post-analytical phase.~~ Percentage of incorrect laboratory test reports and notification of critical results were not evaluated since we didn't have these data

during the study period. Similarly, we didn't measure IQC performance in the analytical phase of testing process.

Conclusions

In this study, there were high rates of TB culture and viral load specimen rejection due to centrifugation problems and the use of wrong specimen containers. Equipment down time and reagent stock out at the national level was the main causes of EID test interruption. The trend of TAT of viral load and EID tests were longer than the targeted average TAT. Hence, training of sample collectors, functional equipment maintenance systems and improved supply chain management are recommended for continuous quality improvement in addition to conducting a large scale study to identify more factors.

Acknowledgments

The authors would like to acknowledge the Amhara Public Health Institute for cooperation to use the database and review records.

References

1. Plebani M, Sciacovelli L, Aita A. Quality Indicators for the Total Testing Process. Clin Lab Med 2017; 37: 187–205.
2. Rin G. Pre-analytical workstations as a tool for reducing laboratory errors. Journal of Medical Biochemistry. 2010; 29 (4):315-24.
3. Hammerling JA. A review of medical errors in laboratory diagnostics and where we are today. Laboratory medicine. 2012; 43 (2):41-4.

4. Kaushik N, Green S. Pre-analytical errors: their impact and how to minimize them. MLO Med Lab Obs. 2014; 46 (5):22-4.
5. O'Connor JD. Reducing post analytical error: perspectives on new formats for the blood sciences pathology report. The Clinical Biochemist Reviews. 2015; 36(1):7.
6. Walz SE, Darcy TP. Patient safety & post-analytical error. Clinics in laboratory medicine. 2013; 33 (1):183-94.
7. ISO 15189. Medical laboratories: requirements for quality and competence. Geneva, International Organization for Standardization, 2012.
8. Jegede FE, Mbah HA, Aminu M, Yakubu TN, Torpey K. Evaluation of Laboratory Performance with Quality Indicators in Infectious Disease Hospital, Kano, Nigeria. Open Journal of Clinical Diagnostics 2015; 5: 1-9.
9. Krleza JL, Honovic L, Tanaskovic JV, Podolar S, Rimac V, Jokic A.Post-analytical laboratory work: national recommendations from the Working Group for Post-analytics on behalf of the Croatian Society of Medical Biochemistry and Laboratory Medicine. Biochem Med (Zagreb) 2019; 29(2):020502.
10. Epner PL, Gans JE, Graber ML. When diagnostic testing leads to harm: a new outcomes-based approach for laboratory medicine. BMJ Qual Saf 2013; 22:ii6–ii10.
11. Ridderhof JC, van Deun A, Kam KM, Narayanan PR, Aziz MA. Role of laboratories and laboratory systems in effective tuberculosis programmes. Bull World Health Organ. 2007; 85 (5):354-9.
12. World Health Organization. Global tuberculosis report 2019. Available at: https://apps.who.int/iris/bitstream/handle/10665/329368/9789241565714-eng.pdf?ua=1

13. CDC. Ethiopia Country Profile. Available at: https://www.cdc.gov/globalhivtb/where-we-work/Ethiopia.pdf
14. Ethiopian National Accreditation Office (ENAO). http://www.enao-eth.org/facility+detail.php?id=179. Accessed date 12 Dec 2019
15. College of Physicians and Surgeons of British Columbia. Laboratory Medicine Proficiency Testing: Available Proficiency Testing Providers. Available at: https://www.cpsbc.ca/files/pdf/DAP-PT-Available-Providers.pdf
16. Cao L, Chen M, Phipps RA, Del Guidice RE, Handy BC, Wagar EA, *et al*. Causes and impact of specimen rejection in a clinical chemistry laboratory. Clin Chim Acta. 2016; 458: 154-8.
17. Karcher DS, Lehman CM. Clinical Consequences of Specimen Rejection. Arch Pathol Lab Med. 2014; 138: 103-108.
18. Lee YJ, Shin S, Roh EY, Yoon J H, Kim DK, Chung HS, *et al*. Acceptability of Sputum Specimens for Diagnosing Pulmonary Tuberculosis. J Korean Med Sci 2015; 30: 733-736.
19. Rollins G. Examining the Consequences of Rejected Specimens, 2015. Available at: https://www.aacc.org/-/media/Files/Transcripts/Clinical-Laboratory-Strategies/Podcasts/2015/examining-the-consequences-of-rejected-specimens.pdf
20. Dikmen ZG, Pinar A, Akbiyik F. Specimen rejection in laboratory medicine: Necessary for patient safety? Biochemia Medica 2015; 25(3):377–85.
21. Rooper L, Carter J, Hargrove J, Hoffmann S, Riedel S. Targeting Rejection: Analysis of Specimen Acceptability and Rejection, and Framework for Identifying

Interventions in a Single Tertiary Healthcare Facility. J. Clin. Lab. Anal. 2017; 31: e22060.

22. Zinyowera S, Simons S, Maparo T, Mbofana E, Ndlovu Z, Souza MD. Economic evaluation of viral load testing and early infant diagnosis in rural Zimbabwe. https://aidsfree.usaid.gov/sites/default/files/economic_vleid_zim_2016.pdf
23. Addo SA, Abdulai M, Yawson A, Baddoo AN, Zhao J, Workneh N, *et al*. Availability of HIV services along the continuum of HIV testing, care and treatment in Ghana. BMC Health Services Research 2018; 18:739.
24. Mesfin EA, Taye B, Belay G, Ashenafi A, Girma V. Factors affecting quality of laboratory services in health facilities in Addis Ababa, Ethiopia. eJIFCC 2017; 28: 205-223.
25. Petrose LG, Fisher AM, Douglas GP, Terry MA, Muula A, Chawani M, Limula H, Driessen J. Assessing Perceived Challenges to Laboratory Testing at a Malawian Referral Hospital. Am. J. Trop. Med. Hyg. 2016; 94: 1426–1432.
26. PEPFAR VL/EID Community of Practice (COOP). Viral Load and Early Infant Diagnosis (VL/EID) Implementation Subject Matter Experts (ISMEs) Reference Manual, 2019. Available at: http://www.aslm.org/wp-content/uploads/2019/02/VL-EID-ISME-Community-of-Practice-Reference-Manual-COP19-01172019.pdf
27. Thiha S, Shewade HD, Philip S, Aung T, Kyaw NT, Oo MM, *et al*. Factors associated with long turnaround time for early infant diagnosis of HIV in Myanmar. Global Health Action. 2017; 10: 1395657.
28. Wexler C, Cheng A-L, Gautney B, Finocchario-Kessler S, Goggin K, Khamadi S, et al. Evaluating turnaround times for early infant diagnosis samples in Kenya from

2011-2014: A retrospective analysis of HIT System program data. PLoS ONE 2017; 12 (8): e0181005.

29. Minchella PA, Chipungu G, Kim AA, Sarr A, Ali H, Mwenda R, et al. Specimen origin, type and testing laboratory are linked to longer turnaround times for HIV viral load testing in Malawi. PLoS ONE 2017; 12(2): e0173009.

Tables

Table 1. Quality indicators established in APHI, 01 January to 30 September 2019

SN	Quality indicators	Target
1.	Equipment down time	Maintained within 5 days
2.	Stock outs	0%
3.	Specimen rejection	Less than 2%
4.	EQA PT performance	≥80%
5.	TAT	90% of tests within defined TAT
6.	IQC performance	100%
7.	Contamination rate	<5% for TB culture
8.	Error rate	<5% for GeneXpert
9.	Service interruption	0%

EQA: external quality assessment; IQC: internal quality control; PT: proficiency testing; SN: serial number; TAT: turnaround time; TB: tuberculosis

Table 2. Monthly test statistics, APHI, 01 January to 30 September 2019

Tests	2019									Total	
	Jan	Feb	Mar	Apr	May	Jun	Jul	Aug	Sep	Done	Rejected
Viral load	2571	2710	3545	2018	2903	2304	3340	2630	2496	24517	106
EID	85	179	0	0	271	87	85	90	65	862	0
TB culture	77	69	87	77	91	32	122	54	85	694	8
GeneXpert TB	4	7	9	60	47	25	56	45	46	299	1
**Total**	**2737**	**2965**	**3641**	**2155**	**3312**	**2448**	**3603**	**2819**	**2692**	**26,372**	**115**

EID: exposed infant diagnosis; TB: tuberculosis

Table 3. Specimen rejection rates of tests at APHI reference laboratories, January to September 2019

	2019									
Tests	Jan	Feb	Mar	Apr	May	Jun	Jul	Aug	Sept	Total
Viral load	9(0.35%)	0	5(0.1%)	0	1(0.03%)	0	5 (0.15%)	25(0.94%)	61 (2.4%)	106 (0.43%)
EID	0	0	ND	ND	0	0	0	0	0	0
TB culture	1(1.3%)	2(2.8%)	0	0	0	0	0	0	5 (5.5%)	8(1.14%)
GeneXpert	0	0	0	0	0	0	0	0	1(2.1%)	1(0.33%)

EID: exposed infant diagnosis; TB: tuberculosis; ND: not done

Table 4. Stock out, equipment down time, and service interruption days APHI, January to September 2019.

Tests									
	Jan	Feb	Mar	Apr	May	Jun	Jul	Aug	Sept
Viral load	0	0	0	0	0	0	0	0	0
EID	0	0	Yes/26 days	Yes/28 days	0	0	0	0	Yes/15 days
TB culture	0	0	0	MGIT yes/15	MGIT yes/31	MGIT yes/31	MGIT yes/31	MGIT yes/31	MGIT yes/31
GeneXpert TB	0	0	0	0	0	0	0	0	0

EID: exposed infant diagnosis; MGIT: Mycobacteria growth indicator tube; TB: tuberculosis

Table 5. Proportion of out of turnaround time of patient results in APHI reference laboratories, January to September 2019

Tests	2019								
	Jan	Feb	Mar	Apr	May	June	Jul	Aug	Sept
Viral load (%)	15.0	12.8	0.0	0.0	18.6	0.0	0.0	12.6	81.5
EID (%)	ND	18.4	ND	ND	34.5	50.6	67.4	100.0	100.0
TB culture (%)	2.1	3.4	0.0	0.0	1.6	0.0	0.0	0.0	10.8
GeneXpert TB (%)	0.0	0.0	0.0	3.7	6.8	15.4	7.3	6.8	6.8

EID: exposed infant diagnosis; ND: not done; TB: tuberculosis

Table 6. Average monthly TAT of HIV and TB related laboratory tests, APHI, January to September 2019

Tests	2019									
	Jan	Feb	Mar	Apr	May	June	Jul	Aug	Sept	
Viral load/ day	12.7	13	7.6	8.9	21.8	21.9	19.2	19.1	24.1	16.5
EID/day	24	23.4	ND	ND	45.6	30.6	24.3	32.8	29.3	30.0
TB culture/ day	50	43.3	48.7	49.8	53.4	51.6	63	65.7	62.8	54.3
GeneXpert TB /day	4.0	3.6	1.0	2.0	2.9	1.6	1.6	2	2.3	2.3

EID: exposed infant diagnosis; ND: not done; TB: tuberculosis

Figures

Fig. 1. Trend of GeneXpert error rates in APHI from January to September 2019

Fig. 2. Trend of TB culture contamination rates in APHI from January to September 2019

 Click here to access/download;Figure;Fig. 1.tif

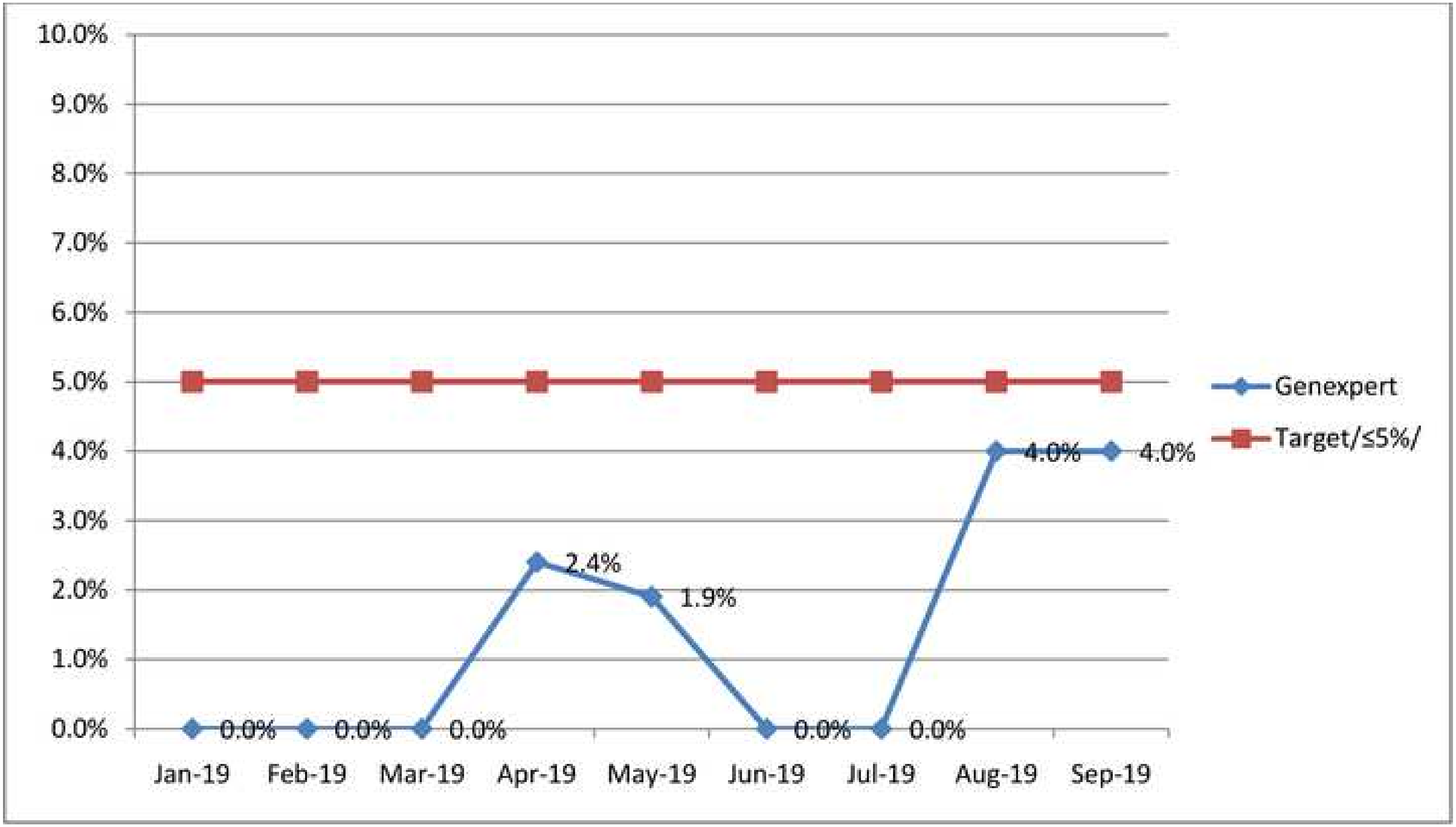

 Click here to access/download;Figure;Fig. 2.tif

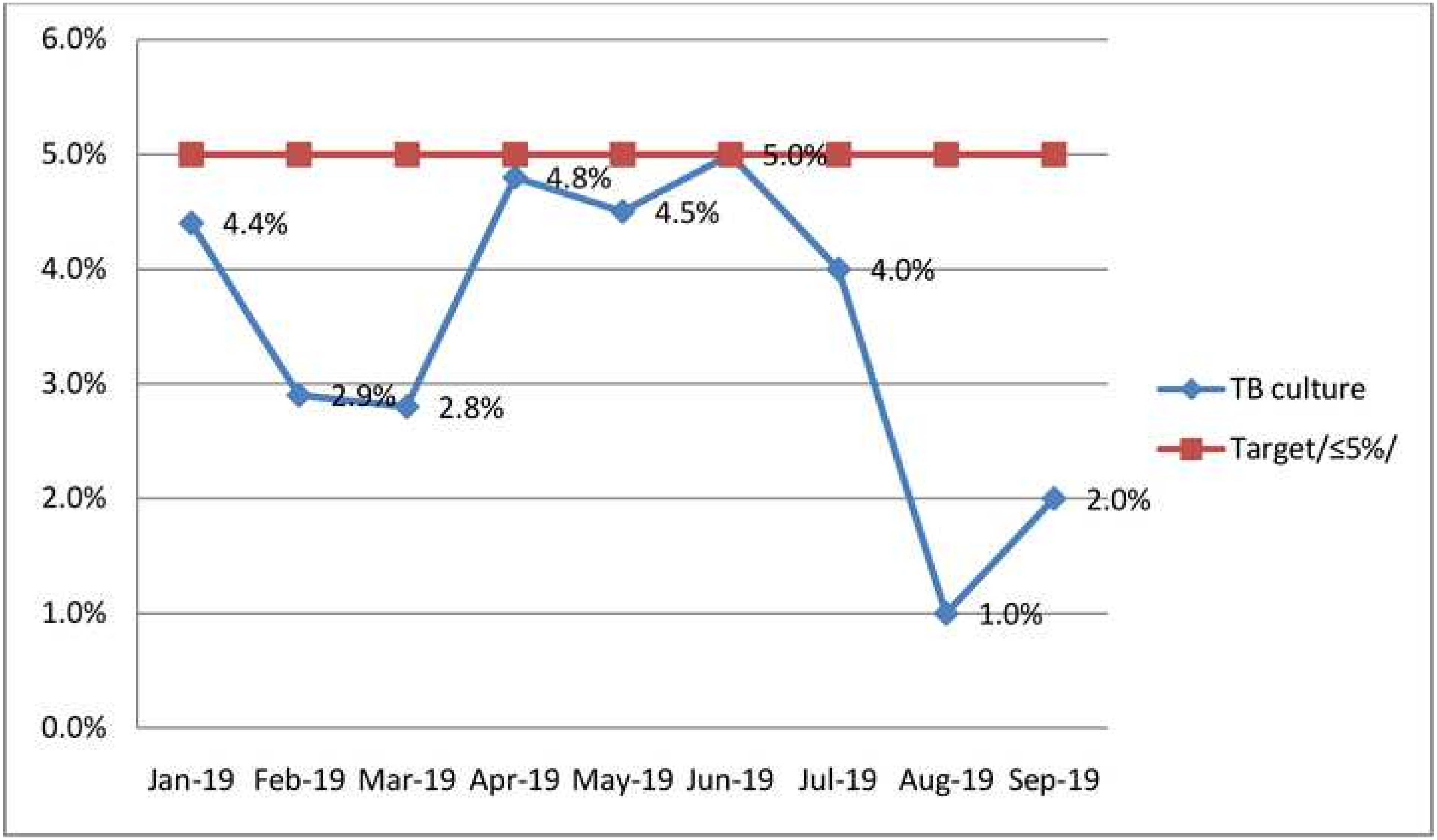

Evaluation of continuous quality improvement of tuberculosis and HIV diagnostic services in Amhara public health institute, Ethiopia

Melashu Balew Shiferaw[1]*, Abay Sisay Misganaw[2]

[1]AResearch and Technology Transfer Directorate, Amhara Public Health Institute, P.O.Box 447, Bahir Dar, Amhara, Ethiopia

[2]Department of Medical Laboratory Sciences, College of Health Sciences, Addis Ababa University, Addis Ababa, Ethiopia

*Corresponding Author

E-mail: bmelashu@gmail.com (MBS)

Abstract

Background: Unreliable laboratory results lead to unnecessary tests, procedures or treatments which may harm patient. Continuous quality improvement (CQI) is a useful objective tool to improve processes and services. The use of quality indicators that meet requirements for effectiveness is an important quality improvement tool. However, the quality of critical aspects of pre-examination, examination, and post-examination processes are not evaluated in Ethiopia including our setting. Hence, this study aimed to assess the performance of continuous quality improvement of TB and HIV laboratory tests in the Amhara Public Health Institute (APHI).

Methods: A cross-sectional study was conducted to evaluate the continuous quality improvement of advanced TB and HIV related laboratory tests in APHI from 01 January to 30 September 2019. Viral load, exposed infant diagnosis (EID), GeneXpertgenexpert and TB culture quality indicators data were used as a quality improvement tool and evaluated in comparison to established targets. Data were extracted from excel database and record review of patient information, and entered and analyzed using SPSS V20 software.

Results: A total of 26,487 samples were received from 01 January to 30 September 2019. The overall specimen rejection rate was 0.43% (115/26,487). Specifically, viral load and TB culture had 0.43% and 01.9214% rejection rates, respectively. The highest monthly rejection was documented for TB culture (5.3%) and viral load (2.4%) in September 2019. Centrifugation problems (46.1% [53/115]) and the use of wrong container (40.9% [47/115]) were the main reasons for the rejections. Moreover, EID test was interrupted for a total of 54 days and 22 days due to reagent stock out and equipment down time, respectively. Similarly, about 82% of viral

load and 100% of the EID tests had long ~~waiting time~~turnaround time (TAT) with an average of 24.1 and 29.3 days respectively in September 2019.

Conclusions: There were high rates of TB culture and viral load specimen rejection, and EID test interruptions. The trend of ~~waiting time~~TAT of viral load and EID tests were longer than the targeted average TAT. Hence, training of sample collectors, functional equipment maintenance systems and supply chain management are recommended for continuous quality improvement.

Keywords: Quality indicator, stock out, specimen rejection, TB, viral load, EID

Introduction

Continuous quality improvement (CQI) is a useful objective tool to improve processes and services. In modern clinical medicine, laboratory tests play an important role in diagnosing, monitoring, and evaluating patient outcomes. Hence, the implementation of performance measurements to evaluate the pre-analytical, analytical and post-analytical stages of the total testing process is therefore needed to maximize the overall testing cycle and the quality of patient care [1].

Pre-analytical error and Post-analytical error data are well documented. Occurrence of the errors at the pre- and post-analytical phases currently appears to be more vulnerable than the analytical phase [2]. Studies revealed that 46%–68.2% of laboratory errors predominated in the pre- analytical phase, and 18.5%–47% errors recorded in the post-analytical phase of the laboratory testing process [3-6].

Formatted: Font: Font color: Black

Formatted: Adjust space between Latin and Asian text, Adjust space between Asian text and numbers

Formatted: Font: 12 pt, Font color: Black

Formatted: Adjust space between Latin and Asian text, Adjust space between Asian text and numbers

The use of quality indicators that meet requirements for effectiveness and harmonization is an important quality improvement tool [~~2~~7]. It can measure how well an organization meets the needs and requirements of users and the quality of all operational processes. Monitoring quality indicators in daily work can reduce laboratory errors and risk to patient safety by identifying problems in all phases of the laboratory process, allowing their correction [8~~3~~].

Stock out of reagents, equipment down time, and sample rejection rates are common pre-analytical phase quality indicators, whereas the performance of proficiency testing (PT) in external quality assessment (EQA) program, internal quality control (IQC) and contamination or error rates are analytical phase indicators. Besides, minimum recommended quality indicators for the post-analytical phase are turnaround time (TAT), the percentage of incorrect laboratory test reports, and notification of critical results [8~~3~~, 9~~4~~].

Laboratories should ~~are expected to~~ deliver accurate, reliable and timely results to customers. When ~~because of~~ the results are compromised ~~test~~ in quality and/ or delayed, ~~results~~ it could have an impact on patient management. As a result, ~~Correct test results may not be timely delivered to the patient. Even the result may be sent after the patient dies. Unreliable results lead~~ the clinician may~~to~~ interpret the results as actionable which, in turn, can lead to unnecessary tests, procedures or treatments which may result in patient harm [10~~5~~].

According to the World Health Organization (WHO)~~WHO~~, drug resistance TB and HIV are global challenges that need quality-assured laboratory tests for better treatment options and control of the burden [11~~6~~]. In addition to the drug resistance burden, if there is poor quality laboratory service, the problem ~~has been~~would be magnified~~ more~~.

Formatted: Adjust space between Latin and Asian text, Adjust space between Asian text and numbers

In Ethiopia, where the estimated proportion of TB cases with Multidrug resistant/ rifampicin-resistant tuberculosis (MDR/RR-TB)~~MDR/RR-TB~~ was16% among previously treated cases and reported a total of 428,472 HIV patients receiving antiretroviral therapy in 2018 [12~~7~~, 13~~8~~], laboratory tests are performed to control drug resistance and monitor disease progression. TB culture is performed to identify treatment failures, exposed infant diagnosis (EID) to identify HIV status in exposed infants, and viral load to monitor confirmed viral failure and estimate disease progression. However, the quality of critical aspects of pre-examination, examination and post-examination processes of ~~such~~ TB and HIV related laboratory services are not evaluated including our setting. Hence, this study aimed to assess the performance of continuous quality improvement using nine months ~~of~~ quality indicators data in the Amhara Public Health Institute (APHI).

Materials and methods

Study design

A cross-sectional study was conducted to evaluate the continuous quality improvement of advanced TB and HIV related laboratory tests in APHI from 01 January to 30 September 2019.

Study setting

APHI is a government public health institute located in Bahir Dar town, Ethiopia at 11°60′N latitude and 37°37′E longitude. The institute has three main directorates (Laboratory, Public Health Emergence management, and research and technology transfer). ~~The APHI~~It provides laboratory diagnostic services including~~for~~ TB (culture for treatment failure, and GeneXpert~~genexpert~~ rifampicin resistance), EID and viral load diagnosis to identify drug resistance for TB, HIV status of infants and confirmed antiretroviral failure, respectively. These

TB and HIV related tests are requested from referring peripheral health facilities in Amhara region that have been networked through a sample referral network. APHI is used as a reference testing center for more than 150 health facilities. All the laboratory tests investigated in APHI including HIV viral load, EID, TB culture and GeneXpert have been accredited by the Ethiopian National Accreditation Office (ENAO) in complying with the ISO 15189:2012 standard since October 2018 [14].

Data collection, and quality indicators and objective of each

In APHI, quality indicators were established to monitor the laboratory performance. EQA program in PT method, contamination rate, error rate specimen rejection rate, TAT, IQC performance, stock out, service interruption and equipment down time were the APHI quality indicators (Table 1). Each target was established by reviewing a minimum of 3 months of data at APHI and also reviewing international literatures.

All of the requested MDR TB, EID and viral load requested tests were included consecutively in the study. Data were collected using a record review of monthly quality indicator reports from January to September 2019. The established quality indicators at the pre-examination phase were equipment down time, stock out, test interruption, and specimen rejection rate.

Equipment down time was set as a quality indicator of APHI virology and bacteriology reference laboratories. All of the equipment should be repaired~~maintained~~ within 5days if there is a failure. Abbott 2000SP and Abbott 2000RT were used for extraction and detection of ribonucleic acid (RNA) of EID and HIV viral load ~~HIV~~ tests. Furthermore, GeneXpert ~~gexpert~~ machine for TB RR testing. TB culture needs 35-37°C incubators. So, we reviewed maintenance records and books of life for each of the equipment in the study period. Besides, no reagent stock out was set

as a quality indicator by APHI~~reagent stock out status was set to be zero~~. Reagent inventory records such as bin card, internal facility request and resupply form (IFRR)~~IFRR~~, and report and requisition form (RRF)~~RRF~~ were reviewed to confirm the occurrence of stock out.

Excel database maintained in the central reception was used to review specimen rejections. Sputum for drug resistant tuberculosis (DRTB) ~~DRTB~~/MDRTB, dried blood spot for EID and blood plasma for viral load samples were submitted at APHI central reception. The entire submitted patient samples were evaluated for sample quality based on pre-set sample rejection criteria. Sputum samples were rejected when there was labeling error, leaking specimen container, sample volume less than 2ml, sample received ~~after~~ 5+ days after ~~of~~ collection, use of the wrong container, specimen containing blood and food ruminants, and temperature exceeding 8°C. The plasma sample was rejected if samples were collected using the wrong container, not-centrifuged, hemolyzed, old (if it was delayed >5 days after collection when transported to APHI at 2-8°C), insufficient volume, labeling error and if not transported below 8°C temperature. Besides, dried blood spot (DBS)~~DBS~~ was rejected when there was hemolysis, insufficient volume of spots, clotting and labeling error. Based on the APHI policy, rejected samples should be communicated to customers and another sample should be recollected for the requested test. The targeted specimen rejection rate was less than 2%.

Proficiency testing performance, contamination rate, error rates, and internal quality control were used for examination phase quality indicators. All of the evaluated tests (TB culture, GeneXpert~~genexpert~~, EID, and viral load) participated in proficiency testing from ~~one~~ Oneworld ~~accuracy~~Accuracy, which is an approved EQA PT ~~PT~~ provider. Panels of samples prepared from the PT provider and sent to APHI. In APHI, the PTs were diagnosed as patient samples and results were submitted online on the Oneworld Accuracy website. Proficiency testing

performance of 80% and above was the pass mark acceptable results~~,~~ [15]. Regarding contamination rate and error rates, less than 5% ~~contamination rate and error rates less than 5%~~target ~~were~~ was set as quality indicators by APHI. Applicable test records were reviewed to evaluate examination phase performances.

Moreover, turnaround time was a post-examination phase quality indicator that 90% of tests should be released within defined turnaround time. TAT of 10 days was targeted for viral load and EID tests. TB culture test TAT was set to be 48 days for each negative result and 64 days for each positive result. Besides, GeneXpert~~genexpert~~ test targeted TAT was 2 days. The TAT was calculated from login and log out of records of each test achieved at the excel database of the central reception. Hence, the ~~waiting time~~TAT of each test was evaluated based on established targets of the institute (Table 1).

Data review, cleaning and data Analysis

The data were entered and analyzed using SPSS V20 software. Selected quality indicators were compared with the defined target and rated as good if complied or not good if not complied with the established target.

Ethical Consideration

Regarding ethical issue, the data were not collected from patients directly since the specimens were collected from the peripheral health facilities and sent to the testing center through a referral network. ~~It was quality indicator reports, record review and from the APHI excel database. So, no need of consent from study participants.~~ Before data collection, ~~E~~ethics approval and official permission letter (Reference number: HRTT/03/139/2018) ~~were~~ was obtained from the APHI research and technology transfer directorate to use patient records from

the APHI excel database, ~~the database~~ quality indicator reports and record review of different quality related records in the laboratory. And the Research and Technology Transfer Directorate waived the requirement for informed consent.

Results

Test statistics and specimen rejection

In Amhara Public Health Institute, a total of 26,487 HIV and TB related test requests with samples were received from 01 January to 30 September 2019. Of which, 26372 (99.6%) had acceptable quality. Among the acceptable samples, 24517 (93.0%) were blood plasma samples for viral load, 862 (3.3%) were dried blood spot samples for EID, 694 (2.6%) were sputum samples for TB culture and 299 (1.1%) were sputum samples for TB GeneXpert~~genexpert~~ tests (Table ~~1~~2).

A total of 115 were rejected from January to September 2019. The overall specimen rejection rate was 0.43% (115/26,487). Specifically, viral load, TB culture and TB GeneXpert~~genexpert~~ specimens had 0.43% (106/24623), 1~~0~~.14~~92~~% (8/702) and 0.33% (1/300) rejection rates, respectively. High monthly sample rejection was documented for TB culture and viral load in September 2019 with 5.3% (5/95) and 2.4% (61/2557) rejection rates, respectively (Table ~~2~~3).

Centrifugation problems (46.1% [53/115]) and the use of wrong container (40.9% [47/115]) were the main reasons for specimen rejection. Viral load specimens were due to centrifugation problem (53/106), use of wrong container (47/106), insufficient volume (5/106) and hemolysis (1/106). ~~Besides~~Moreover, the old samples (5/8), use of a wrong container (1/8), insufficient volume (1/8) and uncontrolled temperature (1/8) were causes of rejection for TB culture samples.

GeneXpert~~Genexpert~~ sample was rejected because of insufficient volume. During specimen rejection, there was proof of communication records about rejected samples. However, there was no evidence of receipt of another sample for the requested tests.

Test interruption

In this study, test interruption was used as a quality indicator. Mycobacteria growth indicator tube (MGIT)~~MGIT~~ TB tests were stocked out. Even if the backup laboratory was assigned, it was impossible to manage due to the issue is national stock out problem and only the Lowenstein-Jensen (LJ)~~LJ~~ culture method was used. EID testing service was continuously interrupted~~ during the nine months~~. Reagent stock out (HIV1 qualitative control stocked out for two months: 05 March to 28 April 2019) and equipment down time (Abbott 2000rt was failed for seven days in March 2019 and Abbott 2000sp was failed for 15 days since 27 September 2019) were the main causes for the EID test interruption (Table ~~3~~4).

Genexpert error rates and TB culture contamination rates

In the Amhara Public Health Institute, the error rate was used as a quality indicator to follow TB GeneXpert~~genexpert~~ tests and contamination rate as used to follow TB culture. Both of the indicators were targeted to be less than 5%. Interestingly, all of the monthly error rates and contamination rates reported were within the targeted limit that had been established by the institute. TB culture contamination rate ~~was~~ increased from March (2.8%) to June (5.0%) in 2019. Then, it was continuously decreased up to the end of August 2019 (1.0%). Regarding TB GeneXpert~~genexpert test~~, the average error rate over the study period was 1.4%, with monthly error rates ranging 0% - 4.0%~~did not occur from January to March, and in June and July 2019. In~~

~~April, August and September 2019, the error rates were 2.4%, 4.0%, and 4.0%, respectively~~ (Fig. 1 and 2).

Performance of proficiency testing

The laboratories participated in April 2019 in the international ~~proficiency testing (~~PT~~)~~ from ~~one~~ Oneworld ~~accuracy~~Accuracy, which is an approved PT provider. All of the tests participated in the PT program qualified the minimum requirement that the passing mark was 80%. Specifically, viral load, EID and GeneXpert~~genexpert~~ had 100% performance as evaluated in April 2019. Besides, TB culture PT performance was 80%, which fulfilled the minimum passing mark.

Turnaround times

The majority of the tests conducted for TB related laboratory diagnosis had good ~~waiting time~~TAT. TB culture tests were released within the targeted TAT except in September 2019. In September, about 11% of the TB culture tests were not released with the targeted TAT. Minimum and maximum average TAT was 43.3 and 65.7 days documented in February and August 2019, respectively. ~~Interestingly,~~ TB GeneXpert~~genexpert~~ tests were relatively had good ~~waiting time~~TAT in March, June and July 2019. However, the average TAT across the study period was 2.3 days with a range of 1.0 to 4.0 days.

Regarding the viral load tests, the average ~~waiting time~~TAT over the study period was 16.5 days, ~~ranged~~ ranging ~~from~~ 7.6 ~~days in March~~ to 24.1 days~~ in September 2019~~. The ~~waiting time~~TAT ~~was~~ improved from January to July 2019 except May 2019 with 18.6% of requested viral load tests out of the defined ~~waiting time~~TAT. Furthermore, the ~~waiting time~~TAT was longer in August and in September 2019 that 12.6%, and 81.5% of viral load tests were delayed, respectively.

EID tests were not done in January, March, and April. However, the ~~waiting time~~TAT for EID was affected more from time to time. For instance, about 35% of the EID tests were delayed in May 2019. Then, 50.6%, 67.4%, 100.0% and 100% of the tests were out of targeted ~~waiting time~~TAT in June, July, August and September 2019, respectively. Over the nine months that the EID test functional, the minimum ~~waiting time~~TAT was 23.4 days occurred in February 2019 and the maximum ~~waiting time~~TAT was 45.6 days documented in May 2019 (Table 5~~4~~ and~~ 5~~6).

Discussion

In this study, 0.45% of the specimens were rejected. EID and liquid TB culture (MGIT method) tests were interrupted over two months in the study period. Similarly, reagent stock out and equipment down time were occurred this period. About 82% of viral load tests and 100% of the EID tests had an average TAT of 45.6 days in September 2019.

In this study, the overall specimen rejection rate was 0.43%. Specifically, it was 1~~0~~.14~~92~~% for TB culture and 0.43% for viral load tests from January to September 2019~~, tests during the nine months~~. Surprisingly, high monthly sample rejection was documented for TB culture and viral load in September 2019 with 5.3% and 2.4% rejection rates respectively. ~~This~~ Those findings ~~is~~ were higher compared to the 2% monthly quality indicator target established by the institute. Also, the finding is higher than the specimen rejection of 0.26% reported by Cao et al in Huston, United States [~~9~~16]. This leads to a high rate of repeat specimen ~~re~~collection, delay in result availability and high rate of specimen/ test abandonment [~~10,~~ 17~~1~~, 18]. Besides, it will have a delay of care, compromise patient safety, waste ~~resources~~ resources~~wastes~~, patient discomfort, and potential patient complications especially if there are critical values [19~~2~~-~~1~~214]. In the setting, there was no evidence of repeat collection of another sample for the requested tests;

rather there was proof of communication records about rejected samples. Hence, the laboratory should proactively take actions such as training of sample collectors on how to collect sufficient samples and process centrifugation of collected samples, and use appropriate sample collection containers in order to ~~solve~~ avoid rejection of samples referred to the institute from peripheral facilities in Amhara region since our data support that nearly half (46.1%) of the specimens were rejected due to centrifugation problem and 40.9% of the samples were rejected because of use of wrong specimen containers.

Without reliable, affordable, and portable devices, regular testing remains a challenge [~~15~~22]. In this study, the EID testing service was continuously interrupted for a period of over two months in addition to the interruption of liquid TB culture (MGIT method) testing. In this period, it was impossible to send samples to other back up laboratories because there was reagent stock out at the national level that all of the laboratories in Ethiopia couldn’t do the testing. ~~Reagent stock out at the national level and equipment down were the main causes of test interruptions.~~ Likewise, about 93% of the testing centers in Ghana had shortage of EID reagents ~~testing was a major challenge that only 7.4% of the testing centers did the testing due to a shortage of reagents in Ghana~~ [~~1~~23~~6~~]. Similarly, equipment down time was the main cause of test interruptions. Studies ~~done~~ in Addis Ababa Ethiopia and in Malawi also revealed that the major reported factors affecting the provision of quality services were a shortage of resources and equipment failure [~~17, 18~~24, 25]. As a quality improvement plan, the laboratory could reduce interruption of tests through rapid implementation of equipment maintenance service agreement, making back up referring laboratory functional, and building maintenance workshop when there is equipment failure. In addition, the institute should establish a system for international bid for advanced laboratory test reagents and supplies when these items stocked out at national level.

An efficient laboratory workflow ensures that specimens received in the laboratory are tested within the established laboratory turnaround time and results are returned to health care providers and their patients on time. However, specimen backlogs can occur when there is suboptimal workflow in the laboratory network as the result of poor sample quality, reagent or consumable stock out, and equipment breakdown [26~~19~~]. In this study, the ~~waiting time~~TAT of viral load and EID tests were longer. About 82% of viral load tests and 100% of the EID tests had ~~an average~~out of targeted TAT ~~of 45.6 days~~ in September 2019. The finding ~~is~~ was higher than a study done in Myanmar ~~and,~~ where long TAT was ~~seen~~ observed in 69% of the participants [~~20~~27], and in Kenya and Malawi ~~that the~~where EID and viral load tests lasted average TAT of 24.7 and 8 days, respectively [~~21~~28, 29~~2~~]. This could be due to stock-outs of reagents and maintenance issues with the automated PCR testing equipment since our data showed reagent stock out of EID in about 54 days and equipment down time for a total of 22 days in the nine months from January to September 2019. In general, the trend of ~~waiting time~~TAT for viral load and EID showed more delay up to the end of September 2019. Hence, APHI laboratory should work on equipment management and stocking issues to provide timely laboratory result to customers.

Limitations

Reader should be into consideration the following limitation while inferring our result. We only measure TAT to evaluate for the post-analytical phase. Percentage of incorrect laboratory test reports and notification of critical results were not evaluated since we didn't have these data during the study period. Similarly, we didn't measure IQC performance in the analytical phase of testing process.

Conclusions

In this study, there were high rates of TB culture and viral load specimen rejection due to centrifugation problems and the use of wrong specimen containers. Equipment down time and reagent stock out at the national level was the main causes of EID test interruption. The trend of ~~waiting time~~TAT of viral load and EID tests were longer than the targeted average TAT. Hence, training of sample collectors, functional equipment maintenance systems and improved supply chain management are recommended for continuous quality improvement in addition to conducting a large scale study to identify more factors.

Acknowledgments

The authors would like to acknowledge the Amhara Public Health Institute for cooperation to use the database and review records.

References

1. Plebani M, Sciacovelli L, Aita A. Quality Indicators for the Total Testing Process. Clin Lab Med 2017; 37: 187–205.
2. Rin G. Pre-analytical workstations as a tool for reducing laboratory errors. Journal of Medical Biochemistry. 2010; 29 (4):315-24.
3. Hammerling JA. A review of medical errors in laboratory diagnostics and where we are today. Laboratory medicine. 2012; 43 (2):41-4.
4. Kaushik N, Green S. Pre-analytical errors: their impact and how to minimize them. MLO Med Lab Obs. 2014; 46 (5):22-4.

5. O’Connor JD. Reducing post analytical error: perspectives on new formats for the blood sciences pathology report. The Clinical Biochemist Reviews. 2015; 36(1):7.

~~1.~~6. Walz SE, Darcy TP. Patient safety & post-analytical error. Clinics in laboratory medicine. 2013; 33 (1):183-94.

~~2.~~7. ISO 15189. Medical laboratories: requirements for quality and competence. Geneva, International Organization for Standardization, 2012.

~~3.~~8. Jegede FE, Mbah HA, Aminu M, Yakubu TN, Torpey K. Evaluation of Laboratory Performance with Quality Indicators in Infectious Disease Hospital, Kano, Nigeria. Open Journal of Clinical Diagnostics 2015; 5: 1-9.

~~4.~~9. Krleza JL, Honovic L, Tanaskovic JV, Podolar S, Rimac V, Jokic A.Post-analytical laboratory work: national recommendations from the Working Group for Post-analytics on behalf of the Croatian Society of Medical Biochemistry and Laboratory Medicine. Biochem Med (Zagreb) 2019; 29(2):020502.

~~5.~~10. Epner PL, Gans JE, Graber ML. When diagnostic testing leads to harm: a new outcomes-based approach for laboratory medicine. BMJ Qual Saf 2013; 22:ii6–ii10.

~~6.~~11. Ridderhof JC, van Deun A, Kam KM, Narayanan PR, Aziz MA. Role of laboratories and laboratory systems in effective tuberculosis programmes. Bull World Health Organ. 2007; 85 (5):354-9.

~~7.~~12. World Health Organization. Global tuberculosis report 2019. Available at: https://apps.who.int/iris/bitstream/handle/10665/329368/9789241565714-eng.pdf?ua=1

13. CDC. Ethiopia Country Profile. Available at: https://www.cdc.gov/globalhivtb/where-we-work/Ethiopia.pdf

~~8.~~14. Ethiopian National Accreditation Office (ENAO). http://www.enao-eth.org/facility+detail.php?id=179. Accessed date 12 Dec 2019

15. College of Physicians and Surgeons of British Columbia. Laboratory Medicine Proficiency Testing: Available Proficiency Testing Providers. Available at: https://www.cpsbc.ca/files/pdf/DAP-PT-Available-Providers.pdf

~~9.~~16. Cao L, Chen M, Phipps RA, Del Guidice RE, Handy BC, Wagar EA, *et al.* Causes and impact of specimen rejection in a clinical chemistry laboratory. Clin Chim Acta. 2016; 458: 154-8.

~~10.~~17. Karcher DS, Lehman CM. Clinical Consequences of Specimen Rejection. Arch Pathol Lab Med. 2014; 138: 103-108.

~~11.~~18. Lee YJ, Shin S, Roh EY, Yoon J H, Kim DK, Chung HS, *et al*. Acceptability of Sputum Specimens for Diagnosing Pulmonary Tuberculosis. J Korean Med Sci 2015; 30: 733-736.

~~12.~~19. Rollins G. Examining the Consequences of Rejected Specimens, 2015. Available at: https://www.aacc.org/-/media/Files/Transcripts/Clinical-Laboratory-Strategies/Podcasts/2015/examining-the-consequences-of-rejected-specimens.pdf

~~13.~~20. Dikmen ZG, Pinar A, Akbiyik F. Specimen rejection in laboratory medicine: Necessary for patient safety? Biochemia Medica 2015; 25(3):377–85.

~~14.~~21. Rooper L, Carter J, Hargrove J, Hoffmann S, Riedel S. Targeting Rejection: Analysis of Specimen Acceptability and Rejection, and Framework for Identifying Interventions in a Single Tertiary Healthcare Facility. J. Clin. Lab. Anal. 2017; 31: e22060.

~~15.~~22. Zinyowera S, Simons S, Maparo T, Mbofana E, Ndlovu Z, Souza MD. Economic evaluation of viral load testing and early infant diagnosis in rural Zimbabwe. https://aidsfree.usaid.gov/sites/default/files/economic_vleid_zim_2016.pdf

~~16.~~23. Addo SA, Abdulai M, Yawson A, Baddoo AN, Zhao J, Workneh N, *et al*. Availability of HIV services along the continuum of HIV testing, care and treatment in Ghana. BMC Health Services Research 2018; 18:739.

~~17.~~24. Mesfin EA, Taye B, Belay G, Ashenafi A, Girma V. Factors affecting quality of laboratory services in health facilities in Addis Ababa, Ethiopia. eJIFCC 2017; 28: 205-223.

~~18.~~25. Petrose LG, Fisher AM, Douglas GP, Terry MA, Muula A, Chawani M, Limula H, Driessen J. Assessing Perceived Challenges to Laboratory Testing at a Malawian Referral Hospital. Am. J. Trop. Med. Hyg. 2016; 94: 1426–1432.

~~19.~~26. PEPFAR VL/EID Community of Practice (COOP). Viral Load and Early Infant Diagnosis (VL/EID) Implementation Subject Matter Experts (ISMEs) Reference Manual, 2019. Available at: http://www.aslm.org/wp-content/uploads/2019/02/VL-EID-ISME-Community-of-Practice-Reference-Manual-COP19-01172019.pdf

~~20.~~27. Thiha S, Shewade HD, Philip S, Aung T, Kyaw NT, Oo MM, *et al*. Factors associated with long turnaround time for early infant diagnosis of HIV in Myanmar. Global Health Action. 2017; 10: 1395657.

~~21.~~28. Wexler C, Cheng A-L, Gautney B, Finocchario-Kessler S, Goggin K, Khamadi S, et al. Evaluating turnaround times for early infant diagnosis samples in Kenya from

2011-2014: A retrospective analysis of HIT System program data. PLoS ONE 2017; 12 (8): e0181005.

~~22.~~29. Minchella PA, Chipungu G, Kim AA, Sarr A, Ali H, Mwenda R, et al. Specimen origin, type and testing laboratory are linked to longer turnaround times for HIV viral load testing in Malawi. PLoS ONE 2017; 12(2): e0173009.

Tables

Table 1. Quality indicators established in APHI, 01 January to 30 September 2019

SN	Quality indicators	Target
1.	Equipment down time	Maintained within 5 days
2.	Stock outs	0%
3.	Specimen rejection	Less than 2%
4.	EQA PT performance	>80%
5.	TAT	90% of tests within defined TAT
6.	IQC performance	100%
7.	Contamination rate	<5% for TB culture
8.	Error rate	<5% for GeneXpert
9.	Service interruption	0%

EQA: external quality assessment; IQC: internal quality control; PT: proficiency testing; SN: serial number; TAT: turnaround time; TB: tuberculosis

Table ~~1~~2. Monthly test statistics, APHI, 01 January to 30 September 2019

	2019										
										Total	
Tests	Jan	Feb	Mar	Apr	May	Jun	Jul	Aug	Sep	Done	Rejected
Viral load	2571	2710	3545	2018	2903	2304	3340	2630	2496	24517	106
EID	85	179	0	0	271	87	85	90	65	862	0

TB culture	77	69	87	77	91	32	122	54	85	694	8
GeneXpert~~Expert~~ TB	4	7	9	60	47	25	56	45	46	299	1
**Total**	**2737**	**2965**	**3641**	**2155**	**3312**	**2448**	**3603**	**2819**	**2692**	**26,372**	**115**

EID: exposed infant diagnosis; TB: tuberculosis

Table ~~2~~3. Specimen rejection rates of tests at APHI reference laboratories, January to September 2019

	2019									
Tests	Jan	Feb	Mar	Apr	May	Jun	Jul	Aug	Sept	Total
Viral load ~~(%)~~	9(0.35%)	0	5(0.1%)	0	1(0.03%)	0	5 (0.15%)	25(0.94%)	61 (2.4%)	106 (0.43%)
EID ~~(%)~~	0	0	ND	ND	0	0	0	0	0	0
TB culture ~~(%)~~	1(1.3%)	2(2.8%)	0	0	0	0	0	0	5 (5.5%)	8(1.14%)
GeneXpert~~Gen expert (%)~~	0	0	0	0	0	0	0	0	1(2.1%)	1(0.33%)

EID: exposed infant diagnosis; TB: tuberculosis; ND: not done

Table ~~3~~4. Stock out, equipment down time, and service interruption days APHI, January to September 2019.

Tests									
	Jan	Feb	Mar	Apr	May	Jun	Jul	Aug	Sept
Viral load	0	0	0	0	0	0	0	0	0
EID	0	0	Yes/26 days	Yes/28 days	0	0	0	0	Yes/15 days
TB culture	0	0	0	MGIT yes/15	MGIT yes/31	MGIT yes/31	MGIT yes/31	MGIT yes/31	MGIT yes/31
GeneXpert~~Genexpert~~ TB	0	0	0	0	0	0	0	0	0

EID: exposed infant diagnosis; MGIT: Mycobacteria growth indicator tube; TB: tuberculosis

Table ~~4~~5. Proportion of out of ~~T~~turnaround time of patient results in APHI reference laboratories, January to September 2019

Tests	2019								
	Jan	Feb	Mar	Apr	May	June	Jul	Aug	Sept
Viral load (%)	15.0	12.8	0.0	0.0	18.6	0.0	0.0	12.6	81.5
EID (%)	ND	18.4	ND	ND	34.5	50.6	67.4	100.0	100.0
TB culture (%)	2.1	3.4	0.0	0.0	1.6	0.0	0.0	0.0	10.8
GeneXpert~~Genexpert~~ TB (%)	0.0	0.0	0.0	3.7	6.8	15.4	7.3	6.8	6.8

EID: exposed infant diagnosis; ND: not done; TB: tuberculosis

~~ND: not done~~

Table ~~5~~6. Average monthly ~~waiting time~~TAT of HIV and TB related laboratory tests, APHI, January to September 2019

Tests	2019									
	Jan	Feb	Mar	Apr	May	June	Jul	Aug	Sept	
Viral load/ day	12.7	13	7.6	8.9	21.8	21.9	19.2	19.1	24.1	16.5
EID/day	24	23.4	ND	ND	45.6	30.6	24.3	32.8	29.3	30.0
TB culture/ day	50	43.3	48.7	49.8	53.4	51.6	63	65.7	62.8	54.3
GeneXpert~~Genexpert~~ TB /day	4.0	3.6	1.0	2.0	2.9	1.6	1.6	2	2.3	2.3

Formatted Table

EID: exposed infant diagnosis; ND: not done; TB: tuberculosis

~~ND: not done~~

Figures

Fig. 1. Trend of GeneXpert~~genexpert~~ error rates in APHI from January to September 2019

Fig. 2. Trend of TB culture contamination rates in APHI from January to September 2019

'Response to Reviewers'.

Dear editor, thank you so much for mailing the comments. We would like to thank the reviewer’s, really well appreciated critics and we learn a lot from the review, and also our work now has been improved much from their comments. The following is our point by point response:

Journal Requirements:

In the ethics statement in the manuscript and in the online submission form, please provide additional information about the patient records/samples used in your retrospective study. Specifically, please ensure that you have discussed whether all data/samples were fully anonymized before you accessed them and/or whether the IRB or ethics committee waived the requirement for informed consent. If patients provided informed written consent to have data/samples from their medical records used in research, please include this information.

Response: thank you, now detailed ethics statement has been included in the manuscript and in the online submission form.

Reviewers' comments:

Reviewer #1:

Abstract:

Background:

• The focus of this study is continuous quality improvement (CQI) assessment using specific laboratory indicators but information on CQI not provided.

Response: Thank you, now it has been provided in the revised manuscript

• The established target was your laboratory specific and should be made clear as such in line 30 including specific target for each quality indicators.

Response: Thank you, it has been made clear now since include specific target for each quality indicators in table 1 of the new version manuscript. But, to include in the abstract part it became more detailed and out of word limit. So, table 1 and description in the method part is enough.

• Replace long waiting time with TAT and effect same correction throughout the text for uniformity and consistency of terminology.

Response: thank you, it has been replaced throughout the text

Methods:

• Line 26 through 30 should contain information of number of facilities and level of health care if such data is available. This would help for another level of analysis to improve the work further. How was the data collected, reviewed cleaned and analyzed was not stated.

Response: thank you, now we have stated how the data was collected, reviewed cleaned and analyzed. However, information of number of facilities and level of health

care data was not available to include as suggested.
• Line 38 stated that ‘’EID had a long waiting time in September 2019’’. What specific time are you referring to?. Include the exact data for the TAT.
Response: thank you, now it has been described. The specific time was 29.3 days as described in the new version manuscript.

Introduction:
• The authors may wish to consider this statement Continuous quality improvement (CQI) is a useful objective tool to improve processes and services and linking it with line 45 through 48 and ensuring effective flow and synchronization. Pre-analytical error and Post-analytical error data are abundant and well documented and would be important to include them in the introduction.
Response: : thank you, pre-analytical error and Post-analytical error data has been included them in the introduction

• Line 54 replace ‘’equipment down’’ with equipment down time and do same throughout text and include internal quality control (IQC) after proficiency testing in line 55.
Response: thanks, now it has been corrected as the reviewer’s advice.
• Replace “has been magnified more’’ with would become magnified in line 66 and expunge such and of in line 74 and 76 respectively.
Response: thanks, now it has been corrected as the reviewer’s advice.
Materials and Methods:
Authors may wish to consider subheading using the following guide for easy readability:
1. Study Design and ensure the actual study design was well stated since data was retrospective
Response: thanks, now it has been corrected
2. Setting should be well describe including longitude and latitude of the study facility
Response: Now it has been well described as shown in the setting part of the method section
3. Data Collection
Response: thanks, now it has been revised
4. Quality indicators and objective of each
Response: thanks, now it has been revised and we include it together with data collection part
5. Data review, cleaning and data Analysis
Response: thanks, now it has been revised
6. Ethical Approval or Ethical Consideration
Response: thanks, now it has been revised
7. Table for quality indicator and threshold or target for the laboratory

Response: Thank you, now the table has been included as the reviewer's advice.
Line 91 replace maintained with repair or fix and do same for gexpert with GeneXpert in line 93.
Response: thanks, now it has been corrected
Please define all abbreviations such as RR, RRF, RRF and DBS in text before they can be use.
Response: thanks, now it has been revised
What of Internal quality control monitoring threshold or target for qualitative data such as viral load testing?
Response: Thanks, it is 100%. It is described in table 1 of the new version manuscript.
Results Section:
• You may wish to consider including characteristics of facilities and various level of health cares including ownership (private and public) in the result section. Include percentage in parenthesis for each absolute number from lie 132 through 137.
Response: Thanks, now we have included percentage in parenthesis for each absolute number from line 132 through 137. However, we couldn't get data for characteristics of health facilities to include as the reviewers recommendation.
• Ensure you do not repeat results in tables for example use term like about half or close to half or one-third etc.
Response: Thank you for the comment; it has been revised according to the reviewers advice.
• Between line 160 and 167 ensure that text are presented as they are arrange in heading and remove was in line 164 after rate. Replace March (2.8%) to June (5.0%) in 2019 with what you have in line 164.
Response: Thank you, now corrections have been made based on the reviewer comments.
• Please replaced waiting time with TAT with waiting time throughout text see Line 175 through 190.
Response: Thank you, now waiting time has been replaced with TAT in the revised version manuscript.
• What of GeneXpert Data?
Response: Now, we specified in the result section under TAT in addition to table 5
• In line 179 replace interestingly with However and expunge was in 181 after TAT, include 2019 after May in line 181 and same in line 189 after February.
Response: Thank you, now it has been corrected

Discussion:
• Authors may wish to consider major findings summary as the first paragraph and discussion of results chronologically as presented in result section in next paragraphs thereafter

Response: Thanks, now we add summary of results in the first paragraph of the discussion section as the reviewer's advice.

• Include period of months in line 193 and replace This finding and is in line 195 with Those findings and was.

Response: Now, it has been corrected

• Where was 0.26% reported by Cao et al., in line 197 and modify sample recollection with repeat specimen collection in line 197 and any other place recollection of sample appear in text

Response: Thanks, it was in Huston, United States. And recollection has been modified based on the reviewer's advice.

• In line 199, include compromise before patient safety and modify resources wastes to waste resources.

Response: Thank you very much; it really helps us to improve our paper. And it has been modified accordingly.

• In line 203 replace sample collectors with Phlebotomy and do so through text and replace sole in line 205 with avoid and include nearly half in line 2016 before 46.1% and include specimen before containers in line 207

Response: Thank you; all recommendations have been corrected in the new version manuscript except Phlebotomy that it better to use as it's, because the term phlebotomy more related to blood collections, but in our study, we have more than blood sample like sputum.

• In line 209 include for a period of after interrupted and remove in the study after month.

Response: Thank you; now it has been modified

• The sentence in line 212 to 213 require modification and done after studies should be expunge

Response: Thank you; now it has been modified

• In line 222 replace is with was and in line 223 remove and replace observed or recorded with seen and line 224 that the should be replace with where and include period after months in 227.

Response: Now, it has been corrected

Conclusion:

Effect on necessary correction pointed out in discussion in conclusion include specimen in line 231 before container and time after down and phlebotomy in line 233 to replace samples collectors and include improved in line 234 after and before supply.

Response: Now, it has been corrected

Tables and Figures

• Table 1 Epert TB should read GeneXpert TB and include Abbreviations Definition under table
Response: Now, it has been corrected
• Table 2 inlcude n(%) for each test and include Abbreviations and Definition
Response: Response: Now, it has been corrected
• Include figure title under each Chart for Line Graph for Fig 1 and Fig 2 respectively
Response: Thank you for the comment. The journal requirement describes to provide figures in separate and title to include in the main manuscript. That is why we didn't put under each figure. The editor may put it under the graph during publication.

Reviewer #2:
Introduction
1. Lines 54-58: You mention that "recommended quality indicators for the post-analytical phase are turnaround time (TAT), the percentage of incorrect laboratory test reports, and notification of critical results," yet you do not measure or report on incorrect laboratory results or notification of critical results. Do you have these data? If not, consider adding to the limitations.
Response: Thank you, comment accepted and it has been described as limitation at the end of the discussion part
2. Lines 59-62: Confusing sentence, consider revising
Response: Thank you, now it has been revised to avoid the confusion.

3. Lines 68-71: The statistics for MRD/RR-TB and ART use seem out of place here and hard to contextualize in terms of laboratory testing requirements. Would be more useful to know – at a national level – how many patients require each of these tests annually.
Response: Thank you. The statistics described was to give background information how much of the patients suffer from HIV and TB related health problems at national level including our setting. All of the listed statistics require quality assured laboratory results. So, we believe this information is important and has not been deleted.

Methods
4. Line 111: Should be "Oneworld Accuracy", please ensure correct spelling and capitalization. Also suggest citing
Response: Thank you, now it has been corrected and cited.

5. Line 93: GeneXpert spelled incorrectly.
Response: Thank you, now it has been corrected

6. Lines 89: How does the indicator “test interruption” differ from equipment downtime and stock out?
Response: Thank you, it differs because the lab uses back up referring laboratories to avoid test interruption when there is either equipment downtime or stock out.
7. Line 95: Unclear to reader what “Besides, reagent stock out status was set to be zero” means.
Response: now it has been modified. It means no reagent stock out was set as a quality indicator by APHI.

8. Line 102: Suggest rephrasing to say, “sample received 5+ days after collection…”
Response: Thank you, now it has been rephrased as the reviewer’s advice

9. Line 104, “old” plasma samples – please quantify what how old is “old”
Response: Thanks, it was said old plasma samples if it was delayed >5 days after collection when transported to APHI at 2-8oC. Now it has been included in the new manuscript.

10. Lines 109-114, please describe in more detail what proficiency testing entails.
Response: Now it has been more entailed and included in the new version manuscript.
11. Lines 112-118: How were these targets (PT 80%, contamination <5%, error <5%, TAT for each test) established?
Response: Thanks, now it has been included in table 1 of the new version manuscript

Results
1. The presentation of results is difficult to follow. I would suggest presenting the all of results for each test separately and then moving on to the next test, keeping the same format and organization for the presentation of results of each test. For example, discuss TB culture first and talk about:
a. Pre-examination indicators:
i. total number of samples for culture TB
ii. rejection rate for culture TB and reasons for rejection
b. Examination phase indicators:
i. Test interruption for culture TB & reasons
ii. Error/contamination rates and reasons for culture TB
c. Post examination phase indicators:
i. TAT for culture TB
Then repeat this sequence of results presentation for GeneXpert, EID, and VL.
Response: Thank you for the comments. But when we tried to rearrange based on the suggested sequence of results, it became more complicated and difficult to describe repeated quality indicators. So, we prefer the current sequence of results presentation.

2. The inclusion of data per month complicates the results, without adding much benefit, since there does not seem to be a temporal trend in indicators. I would suggest removing references to months when specific rates were achieved. Instead, the results could be simplified by first giving the average across the entire study period and then giving the low to high range, rather than specify the value per month.
a. For example, Lines 165-167 could be simplified to “Regarding TB GeneXpert, the average error rate over the study period was XX%, with monthly error rates ranging from 0%-4%.”
Response: now it has been simplified based on the reviewer’s advice.

3. No examination phase measures for HIV viral load testing are discussed.
Response: Thank you, it has been already discussed in the result section under proficienc testing performance stating “All of the tests participated in the PT program qualified the minimum requirement that the passing mark was 80%. Specifically, viral load, EID and GeneXpert had 100% performance as evaluated in April 2019”. But IQC was not discussed since we didn’t evaluate it and we put as limitation in the new version manuscript.

4. average TAT across the study period was not specified for each test, rather, monthly rates were given, which unnecessarily complicates the results.
Response: Thank you, now it has been included in the result section and in table 5.

5. TAT for GeneXpert RR tests are not specified, other than “good”.
Response: Now, it has been specified in the new version manuscript

6. Tables 4 and 5. What is the difference between turnaround time and waiting time?
Response: Thank you so much, comments help us to improve the paper. Table 4 describes the proportion of out of TAT patient results in APHI, and Table 5 describes the average TAT of tests. In the new version manuscript, the title of table 4 has been modified as “Proportion of out of turnaround time of patient results in APHI reference laboratories, January to September 2019”

7. Table 4: I’d suggest a title that makes it clearer that the numbers presented are rates of samples that achieved TAT targets. As is, that is not clear.
Response: Thank you the title now has been cleared. Yes, rates of samples that achieved TAT targets

Discussion
• The authors miss an opportunity to discuss recommendations to address the gaps that their data highlights. The only recommendation offered discusses the need for training to address sample rejection issues (lines 202-206). While important, this will not help

with machine down, stockouts and long turnaround times – what can be done to improve indicators for these?
Response: Thank you so much, very important view and help to improve our work. Now, recommendations have been included to address the gaps highlighted.

• Please discuss the 2 months where no EID tests were conducted – what happened to the EID samples during the period: were they sent to other labs? Were they held on to and processed once the machine was repaired and reagents replenished – if so, this could explain the increased sample volume and TAT observed in May?
Response: Thank you, the 2 months EID interruption was due to the national stock out occurred in Ethiopia that all of the labs in Ethiopia were not able to test EID. It was impossible to send samples to other laboratories. In addition, equipment was failed in April for seven days as explained in the result section. Maintenance was done after that. No, these issues have been explained in the discussion section.

• Line 221-222: "About 82% of viral load tests and 100% of the EID tests had an average TAT of 45.6 days in September 2019."
Response: Thank you so much, now it has been corrected as "About 82% of viral load tests and 100% of the EID tests had out of targeted TAT in September 2019".

• Suggest adding a limitations section.
Response: thanks, now limitation section has been included in the new version manuscript.

General comments
1. Please define all acronyms at first use. Some that were not accurately defined include: MDR/RR-TB (line 68); IFRR/RRF (line 96); DRTB (line 98), MGIT (line 152), LJ culture (line 154)
Response: thanks, now it has been revised
2. Please be sure to write GeneXpert with correct capitalization pattern
Response: thanks, now it has been revised
