# Supplementary material for: Evaluation of continuous quality improvement of tuberculosis and HIV diagnostic services in Amhara Public Health Institute, Ethiopia
Source: PLoS One. 2020 Mar 19;15(3):e0230532. doi: 10.1371/journal.pone.0230532 (PMC7081999; doi:10.1371/journal.pone.0230532)
Supplement: Attachment — Submitted filename: Response to Reviewers.docx [file pone.0230532.s003.docx]

Response to Reviewers.

Dear editor, thanks for providing us the comments, and also we thank the reviewers. Accordingly, we have addressed requested revisions that the manuscript has been further edited for language based on the recommendations. See the new version manuscript and the tracked change manuscript submitted.

Regards,

Melashu
